# Homologous recombination is essential for DNA damage-induced regeneration of *Drosophila* testis germline stem cells

**DOI:** 10.1242/dev.205514

**Published:** 2026-08-13

**Authors:** Jasmine R. Grey, Salman Hasan, Janelle Bellot, Margaret de Cuevas, Erika L. Matunis

**Affiliations:** Department of Cell Biology, Johns Hopkins University School of Medicine, 725 N. Wolfe Street, Baltimore, MD 21205, USA

**Keywords:** Stem cell niche, p53, Blm, DNA repair, Spermatogenesis, Regeneration

## Abstract

Germline stem cells (GSCs) from mammals to insects preferentially survive ionizing radiation but the underlying mechanisms are not well understood. Here, we use the *Drosophila* testis to study the responses of GSCs to DNA damage in an intact tissue. We find that 75 Gy of ionizing radiation causes widespread DNA damage and rapidly reduces the GSC population by about half with GSCs lost from the niche via detachment rather than apoptosis. The remaining GSCs are functional, repopulating the niche. This series of events requires members of the canonical DNA damage response pathway, Chk2 and p53, to respond to double-strand breaks induced by ionizing radiation. For survival, GSCs do not require the canonical nonhomologous end joining pathway members Ligase4 or Ku70 for DNA repair, but instead cell-autonomously require the homologous recombination factors Rad51 and Blm. In contrast with many other cells, which can use other repair mechanisms, our findings indicate that GSCs preferentially promote accurate DNA repair over error-prone repair, establishing the *Drosophila* testis as a model for studying stem cell DNA repair pathway choice *in vivo*.

## Teaser

**Summary:** The germline stem cells of adult *Drosophila* testes cell-autonomously require canonical DNA damage response machinery and homologous recombination to recover from ionizing radiation and regenerate the tissue.

## INTRODUCTION

Adult stem cells sustain tissue maintenance and recovery by producing daughter cells that remain stem cells or differentiate to continually replenish the tissue (Greenspan et al., 2015). Organisms are challenged by genotoxic stressors that cause differentiated cells to die while stem cells persist (Mandal et al., 2011). Stressors can be endogenous, such as reactive oxygen species or replication stress, or can come from exogenous sources such as ionizing radiation (IR) or chemotherapeutic agents (Helleday et al., 2014). IR is a particularly damaging source of stress as it induces DNA double-strand breaks that cause cell death if left unrepaired (Cannan and Pederson, 2016; Sancar et al., 2004).

The molecular mechanisms for sensing and repairing IR-induced DNA double-strand breaks are highly conserved across species (Clay and Fox, 2021). Double-strand breaks are sensed by phosphoinositide 3-kinase (PI3K)-related kinases or the ataxia-telangiectasia- and RAD3-related (ATR), ataxia-telangiectasia mutated (ATM) or Mre11-Rad50-Nbs1 (MRN) complexes, initializing a signal transduction cascade. This signaling results in cell cycle arrest followed by DNA repair, cell death or senescence through activation of Checkpoint kinases 1 and 2 [Chk1 (Grp in *Drosophila*) and Chk2 (Loki in *Drosophila*)] and the tumor suppressor factor p53. The two predominant pathways used to repair double-strand breaks are homologous recombination (HR) and canonical/classical nonhomologous end joining (c-NHEJ). Which pathway(s) is used by the cell depends on its cell cycle phase at the time of arrest (Mandal et al., 2011). HR accurately repairs double-strand breaks using the sister chromatid as a template and is therefore available only during the S and G2 cell cycle phases. c-NHEJ ligates the broken ends of DNA together, often resulting in mutations, but is available throughout the cell cycle. The two pathways also require different proteins. HR uses endo- and exonucleases at the double-strand break site but Rad51 (Spn-A in *Drosophila*) specifically performs homologous strand search and invasion to synthesize the broken DNA segment (Chapman et al., 2012). HR also requires the Bloom syndrome helicase protein (Blm), which was initially thought to support the NHEJ pathway based on studies done in *Drosophila* cells in culture (Min et al., 2004). However, in the germ cells of the *Drosophila* adult testis and in mammalian cells, Blm is involved in the dissolution of double-Holliday junctions and catalyzes DNA resection at the break (Johnson-Schlitz and Engels, 2006; Sekelsky, 2017; Zhao et al., 2020). The c-NHEJ pathway in most organisms uses Ku70/80, DNA-PKcs and DNA ligase IV (Lig4) proteins to bind and ligate broken ends of DNA. Ku70 (Irbp in *Drosophila*) and its binding partner Ku80 have high affinity for double-stranded DNA ends, enabling them to bind to the DNA break site and initialize c-NHEJ (Zahid et al., 2021). Lig4 forms a complex with additional repair proteins to perform the final step of c-NHEJ, which is to ligate the broken DNA ends together (Lieber, 2010). *Drosophila* lacks orthologs of DNA-PKcs, but Ku70/Ku80 and Lig4 (DNAlig4 in *Drosophila*) are still used for end joining (Sekelsky, 2017). In *Drosophila*, as in other organisms, redundancies have been built into the system: cells unable to repair DNA double-strand breaks through one pathway are typically able to use another pathway (Johnson-Schlitz et al., 2007; Tan et al., 2023).

Between different stem cell populations, there is much variation in the DNA damage response and choice of double-strand break repair pathway, leading to variation in radiosensitivity (Blanpain et al., 2011; Fabbrizi et al., 2018). In mice, hematopoietic stem cells can survive a dose of irradiation that is sufficient to drive intestinal stem cells into apoptosis; this discrepancy is thought to arise from differences in the expression levels of anti-apoptotic and DNA repair proteins between the two stem cell populations (Blanpain et al., 2011). Upon irradiation, cultured mouse quiescent hematopoietic stem cells use NHEJ for DNA repair; however, if they are forced to proliferate and then irradiated, they use the HR pathway instead (Mohrin et al., 2010). There is also variation in the DNA damage response in germline stem cells (GSCs) from different organisms. For example, *Caenorhabditis elegans* germ cells primarily use HR for DNA repair after irradiation (Clejan et al., 2006) and repress the NHEJ pathway (Deng et al., 2015). However, if the Fanconi anemia pathway is perturbed, then the GSCs specifically use NHEJ instead, likely because Fanconi anemia pathway proteins normally repress NHEJ (Deng et al., 2021). Mouse GSCs spend the majority of the cell cycle in the G1 phase (Ishii et al., 2014). However, HR is unavailable during G1, implicating NHEJ for repair. Indeed, one study suggests that GSCs in the adult mouse testis use an alternative form of NHEJ to repair irradiation-induced double-strand breaks (Rübe et al., 2011). The GSCs of the adult *Drosophila* testis upregulate the anti-apoptotic factor *Drosophila* inhibitor of apoptosis 1 (*Diap1*) to resist radiation-induced cell death (Hasan et al., 2015). However, the primary DNA damage repair pathway(s) used by this GSC population have not yet been studied.

**Fig. 1.**
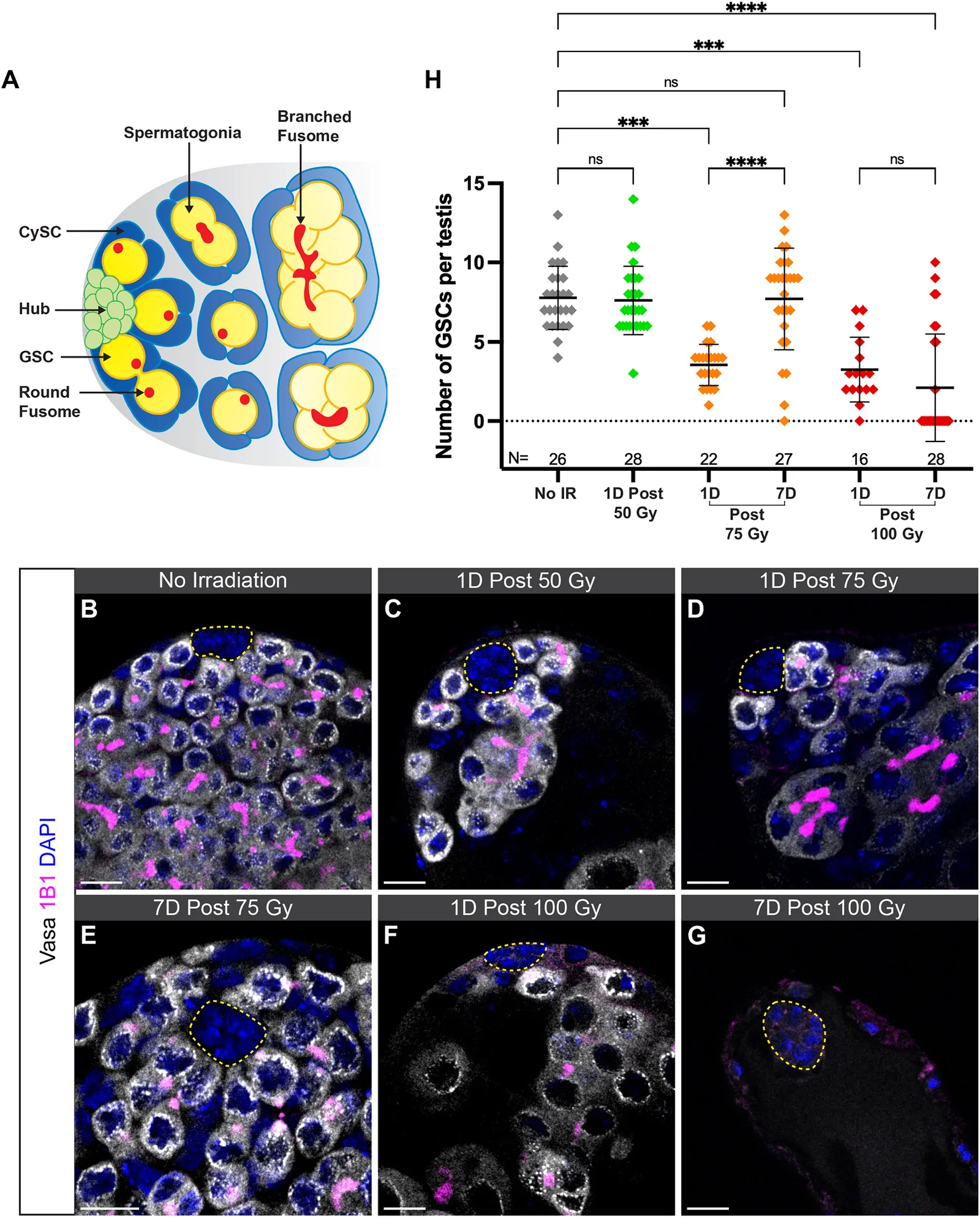
**Reversible reduction of germline stem cell number in the testis niche occurs post 75 Gy irradiation.** (A) Illustration of the *Drosophila* adult testis stem cell niche. Germline stem cells (GSCs; yellow) and somatic cyst stem cells (CySCs; dark blue) are adjacent to the hub (green) at the testis apex. GSCs divide to give rise to gonialblasts, which continue to divide, forming spermatogonia. CySCs divide to form cyst cells, which envelop gonialblasts and their progeny. GSCs were counted as germ cells adjacent to the hub and containing a round fusome (red). (B-G) Confocal sections of control testes immunostained with anti-Vasa (germ cells; white), anti-1B1 (fusomes; magenta) and DAPI (DNA; blue) from non-irradiated flies (B) or 1 or 7 days after exposure to 50, 75 or 100 Gy γ-irradiation as indicated (C-G). The hub is outlined in yellow. Scale bars: 10 μm. (H) Quantification of the data from B-G showing the number of GSCs per testis apex without or with exposure to γ-irradiation as indicated. After 75 Gy, the number of GSCs is significantly reduced, but most testes regain GSCs by 7 days; this radiation dose was used for all subsequent experiments. ***P<0.001, ****P<0.0001 (Kruskal–Wallis test with Dunn's multiple comparisons). ns, not significant. Bars indicate mean±s.d. N=number of testes.

The *Drosophila* gonad is an ideal system in which to study the mechanisms of DNA damage-induced regeneration because it is a well-characterized tissue (de Cuevas and Matunis, 2011). Each adult *Drosophila* testis contains a cluster of somatic niche cells termed the hub that creates a local microenvironment to sustain two stem cell populations: GSCs and somatic cyst stem cells (CySCs) (Fig. 1A). CySCs give rise to differentiated cyst cells, which encapsulate and support the germline. GSCs produce differentiating daughter cells, called gonialblasts, that will execute transit-amplifying divisions to become spermatogonia. The spermatogonia continue differentiating into spermatocytes, undergoing meiosis and eventually developing into sperm. Here, we establish an assay using γ-irradiation to induce significant DNA damage that results in partial GSC loss, allowing us to follow the fate of individual cells and identify the molecular pathways required for regeneration.

## RESULTS

### 75 Gy γ-irradiation is sufficient to induce DNA damage, GSC loss, and regeneration *in vivo*

To test the ability of GSCs to recover from γ-irradiation-induced DNA damage, we first needed to determine a level of whole-body irradiation that causes a reversible loss in the number of GSCs per *Drosophila* testis niche. Previous studies found that loss of spermatogonia can be detected by 24 h after 40 Gy X-irradiation (Welshons and Russel, 1957) or 50 Gy γ-irradiation (Xing et al., 2015). However, irradiation doses up to 50 Gy are not sufficient to cause a significant reduction in GSC numbers (Hasan et al., 2015; Xing et al., 2015). Therefore, we irradiated control males with 50, 75 or 100 Gy and then dissected, fixed and immunostained testes 24 h later and determined the number of GSCs per testis. Consistent with previous results (Xing et al., 2015), we found no significant reduction in GSC number 1 day post 50 Gy irradiation (Fig. 1B,C,H). However, 1 day after exposure to 75 or 100 Gy, the GSC numbers dropped significantly (Fig. 1D,F,H). As expected, loss of spermatogonia was also evident post irradiation at all three doses (Fig. 1C,D,F). To assay directly for DNA damage, we stained testes with γH2AvD antiserum, which labels DNA double-strand breaks in many tissues, including the adult testis, at 1-2 h after irradiation (Delabaere et al., 2017; Madigan et al., 2002). This revealed widespread DNA damage throughout the testis apex that amplified with increasing doses (Fig. S1A-D). Taken together, these results suggest that irradiating flies with 75 or 100 Gy causes enough DNA damage to significantly reduce the number of GSCs remaining in the niche 24 h later.

To determine whether lost GSCs can be replenished over time, we examined testes at 7 days post irradiation. At this time point, the average number of GSCs per testis in males exposed to 75 Gy irradiation was indistinguishable from that in non-irradiated controls (Fig. 1E,H). Additionally, we could no longer detect bright γH2AvD staining at the testis apex at this time point, suggesting a full resolution of DNA damage foci (Fig. S1E-H′). By contrast, after exposure to 100 Gy irradiation, the average number of GSCs remained significantly lower than in non-irradiated controls, with most testes completely lacking GSCs at 7 days post irradiation (Fig. 1G,H). These results are consistent with a historical study reporting a complete loss of spermatogonia and spermatocytes 4-7 days after exposure to 100 Gy X-irradiation (Welshons and Russel, 1957). As GSC numbers declined and then recovered in testes irradiated with 75 Gy, but not 100 Gy, we selected the former dose for all further work: mention of irradiation henceforth indicates this dose.

### DNA damage-induced GSC loss is not due to apoptotic cell death in the niche

**Fig. 2.**
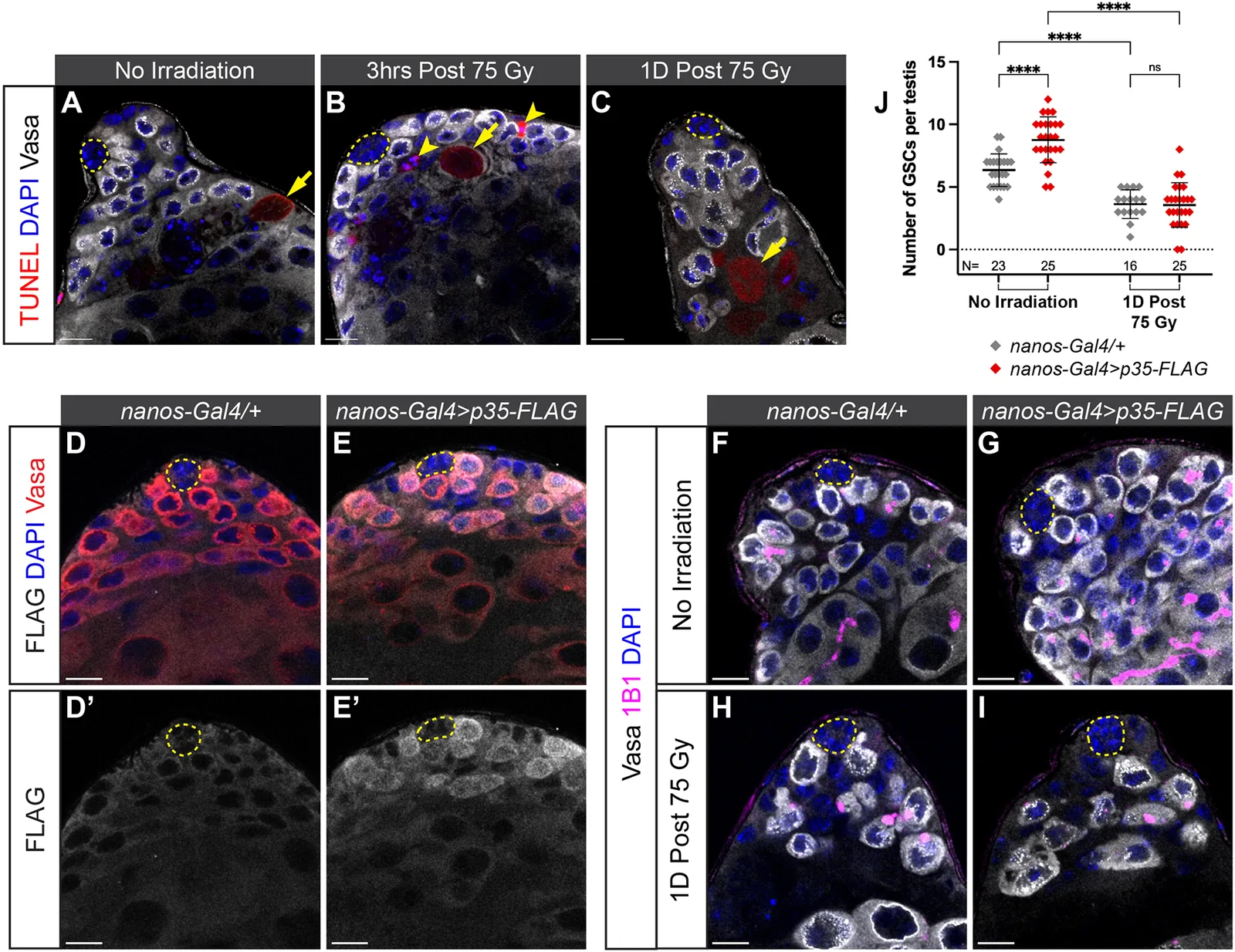
**Apoptosis is not responsible for loss of GSCs from the niche post 75 Gy irradiation.** (A-C) Confocal sections of control testes stained with TUNEL (apoptotic cells; red), anti-Vasa (germ cells; white) and DAPI (DNA, blue) from non-irradiated flies (A) or 3 h (B) or 1 day (C) after exposure to 75 Gy γ-irradiation. TUNEL-positive spermatogonial cysts (yellow arrows) are seen in irradiated and non-irradiated testes. TUNEL-positive somatic cells (yellow arrowheads) are seen in irradiated testes. No TUNEL-positive, Vasa-positive cells in direct contact with the hub (outlined in yellow) are seen in any testes (quantified in Table 1). (D-E′) Confocal sections through the apex of testes immunostained with anti-FLAG (p35 expression; white), anti-Vasa (germ cells; red) and DAPI (DNA; blue). D′ and E′ show FLAG channel alone. p35-FLAG protein levels appear higher in testes overexpressing p35-FLAG (E) than in control testes (D). (F-I) Confocal sections through the apex of testes immunostained with anti-Vasa (germ cells; white), anti-1B1 (fusomes; magenta) and DAPI (DNA; blue). Control testes (*nanos-Gal4/+*) and testes expressing the caspase inhibitor p35 in GSCs and their daughters (*nanos-Gal4>p35-FLAG*) are shown with or without irradiation as indicated. (J) Quantification of the data from F-I. Significant loss of GSCs occurred post-irradiation in control testes and testes overexpressing p35 in GSCs and their daughters, indicating that p35 overexpression does not prevent GSC loss after irradiation. ****P<0.0001 (ordinary two-way ANOVA with Tukey's multiple comparisons test). ns, not significant. Bars indicate mean±s.d. N=number of testes. In all confocal sections, the hub is outlined in yellow. Scale bars: 10 μm.

Since irradiation robustly induces spermatogonial death (Hasan et al., 2015), we speculated that loss of GSCs post irradiation could also be due to cell death. To examine this possibility, we identified dying cells with terminal dUTP nick end labeling (TUNEL), which has been previously used to identify dying germ cells in both non-irradiated and irradiated testes (Hasan et al., 2015; Yacobi-Sharon et al., 2013; Zohar-Fux et al., 2022). In non-irradiated *y w^1118^* testes, we saw no TUNEL-positive cells adjacent to the hub that also expressed the germ cell marker Vasa. However, we did see low levels of dying spermatogonia outside the niche, as expected (Fig. 2A, Table 1) (Hasan et al., 2015). Surprisingly, at either 3 h or 1 day after irradiation, we again saw no TUNEL-positive, Vasa-positive cells adjacent to the hub (Fig. 2B,C, Table 1), suggesting that GSCs do not undergo apoptosis in this location at these time points. This finding was unexpected, since the number of GSCs decreases significantly at this time point (Fig. 1H). Of note, 1 day after irradiation, several testes contained a single TUNEL-positive, Vasa-positive cell just outside of the niche, which was scored as a gonialblast (Table 1). We also saw TUNEL-positive cells lacking the germ cell marker Vasa both adjacent to and farther from the hub at 3 h post irradiation (Fig. 2B); these are likely somatic CySCs or their daughter cells, but they could also be dying germ cells no longer expressing Vasa (Zohar-Fux et al., 2022).

**Table 1.** TUNEL staining is not detected in GSCs after 75 Gy irradiation

	No irradiation	3 h post 75 Gy	1 day post 75 Gy
TUNEL staining in GSCs	0/100 testes	0/39 testes	0/60 testes
TUNEL staining in gonialblasts	0/100 testes	0/39 testes	3/60 testes

Testes were stained as described in Fig. 2. Testes with at least one TUNEL-positive GSC were quantified as a fraction of the total number of testes. Vasa-positive cells in direct contact with the hub were identified as GSCs. TUNEL staining was not seen in GSCs under normal conditions, as expected, and was also not seen in GSCs either 3 h or 1 day post irradiation. TUNEL staining was seen in gonialblasts only at the 1 day post irradiation time point.

As an alternative method to determine whether GSC loss after irradiation is due to cell death, we expressed the *Baculovirus* caspase inhibitor p35, which effectively suppresses apoptosis in *Drosophila* (Hay et al., 1994; Yu et al., 2002), in males recovering from irradiation. We used nanos-Gal4 to drive expression of a p35 transgene tagged with a FLAG octapeptide (*p35-FLAG*) in GSCs and early germ cells, and immunostained testes with FLAG antisera to verify expression of the transgene (Fig. 2D-E′). GSC number was significantly higher in non-irradiated testes with *p35-FLAG* expression than in control (*nanos-Gal4/+*) testes, perhaps due to normal variation in GSC number across genotypes (Malpe et al., 2020). Despite this, there was no difference between the two groups at 1 day post irradiation, suggesting that expression of p35 did not prevent irradiation-induced loss of GSCs (Fig. 2F-J). Overall, our results suggest that GSCs do not undergo apoptosis within the niche after 75 Gy irradiation. This finding is consistent with a previous report of GSC response to DNA damage in the ovary (Ma et al., 2016), but the fate of the missing GSCs in either case remained unknown.

### GSCs detach and are displaced from the niche post irradiation

**Fig. 3.**
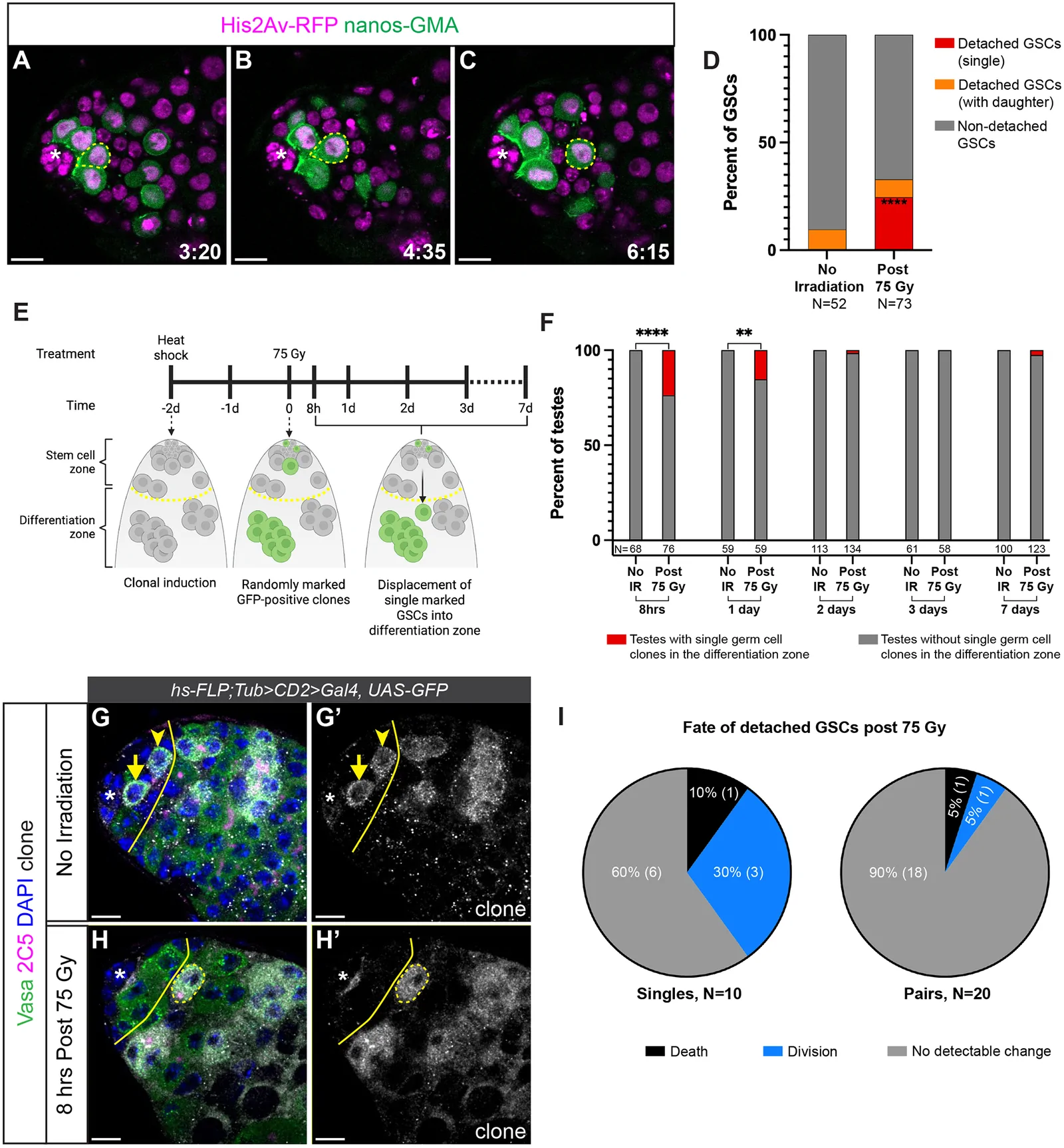
**GSCs leave the niche as single cells post 75 Gy irradiation.** (A-C) Time-lapse confocal sections of a testis apex live imaged 3 h after irradiation with 75 Gy. Nuclei are marked by transgenic expression of Histone 2Av fused to red fluorescent protein (His2Av-RFP; magenta); GSCs and their daughters are marked by *nanos* promoter-dependent expression of GFP fused to the actin-binding domain of Moesin (nanos-GMA; green). Time stamps indicate h:min from the start of imaging. A GSC detaching from the hub is outlined in yellow. The hub is marked with a white asterisk. (D) Stacked bar graph showing the percentage of detached GSCs (single; red) or detached GSCs (with daughter; orange) or remaining attached for the duration of imaging (gray), in irradiated and non-irradiated testes. N=total number of GSCs imaged. ****P<0.0001 (binomial test). (E) Experimental design for identifying single, GFP-marked germ cell clones in the differentiation zone of the testis apex (see Materials and Methods). Created in BioRender by Grey, J., 2026. https://BioRender.com/2yhwf7k. This figure was sublicensed under CC-BY 4.0 terms. (F) Quantification of the data from G-H′ shows a significant increase in the percentage of testes containing single, GFP-marked germ cells in the differentiation zone (red) in irradiated testes compared to non-irradiated controls. Gray bars represent testes without single, marked germ cells in the differentiation zone. Differences at 2, 3 and 7 days are not significant. ****P<0.0001, **P<0.0021 (binomial test). N=total number of testes. (G-H′) Confocal sections through the apex of testes containing GFP-marked clones, immunostained with anti-GFP (white), anti-Vasa (germ cells; green), anti-2C5 (fusomes; magenta) and DAPI (DNA; blue). Solid yellow lines separate the ‘stem cell zone’ (two tiers of cells adjacent to the hub) and the ‘differentiation zone’ (farther from the hub). Clonal marking highlights single germ cells, which are found only in the stem cell zone in non-irradiated testes (yellow arrows in G,G′) and gonialblasts (yellow arrowheads), but also in the differentiation zone in irradiated testes (yellow outline in H,H′). The hub is marked with an asterisk. G′ and H′ show GFP (white channel) only, which marks clones. Scale bars: 10 μm. (I) Pie charts representing the fates detached single GSCs (left) or detached GSC-daughter pairs (right) observed during live imaging of testes beginning 3 h post irradiation. Fates were categorized as death, division or no detectable change. Within each pie fraction is the percentage and the sample number in parentheses. N is the number of GSCs that detached and then displayed each fate during live imaging. Scale bars: 10 μm.

Since we could not detect dying GSCs within the niche after irradiation, we performed *ex vivo* live imaging of testes to visualize GSCs as they were being lost. We imaged testes from flies expressing Histone 2Av fused to red fluorescent protein (His2Av-RFP) to mark nuclei, and the actin-binding domain of Moesin fused to GFP (GMA) under control of the germ cell-specific *nanos* promoter to mark germ cells (Sano et al., 2005). Previous live-imaging analyses of unperturbed testes found that GSCs detach from the hub infrequently and only as interconnected pairs of cells, i.e. while the GSC remains connected to its daughter cell (Sheng and Matunis, 2011). Consistent with prior analyses, we observed infrequent detachment of GSC-daughter pairs in both non-irradiated and irradiated testes, and detaching single GSCs were undetectable in testes from non-irradiated males (Fig. 3D). By contrast, in testes from irradiated males, single GSCs often detached and moved away from the hub: 25% of all GSCs imaged in irradiated testes detached as single cells (Fig. 3A-D, Movie 1). Detached GSCs did not return to the hub or divide for the duration of each imaging session (12.5 h). Together, these results suggest that GSCs are lost after irradiation, not because they are undergoing apoptosis within the niche, but because they detach and displace away from the hub as single cells.

We next turned to *in vivo* clonal marking to capture evidence of GSC detachment in fixed testes, allowing for higher throughput analyses than live imaging. To identify the time point(s) at which GSC detachment might be most frequent, we fixed and stained testes from 2 h to 24 h after irradiation and determined the number of GSCs per testis. GSC numbers began to decline at 4 h post irradiation and were significantly reduced by 6 h compared to non-irradiated controls; from 10 h onward, the average number of GSCs per testis was similar to that at the final 24-h time point (Fig. S2). As the number of GSCs was still declining at 8 h post irradiation, we chose this time point for clonal analysis. We induced low levels of GFP-marked clones in adult males using the heat-shock inducible FLP/FRT ‘flip-out’ system (Struhl and Basler, 1993), which we have used previously to mark cells in the testis (Greenspan and Matunis, 2018). Adult males were heat-shocked to induce clones, allowed to mature for 2 days to establish marking, and then irradiated; testes were then dissected, fixed and immunostained to identify clones (Fig. 3E). In testes from non-irradiated males, single germ cells (GSCs or gonialblasts) are expected near the hub but not farther away in the ‘differentiation zone’ (Lenhart and DiNardo, 2015). Therefore, we searched for single marked germ cells in the differentiation zone, defined here as the region of the testis apex excluding the first two tiers of cells adjoining the hub. As expected, single marked germ cells were not seen in the differentiation zone in non-irradiated testes (Fig. 3F-G′). However, by 8 h after irradiation, they were evident in 24% of irradiated testes (Fig. 3F,H,H′), consistent with the hypothesis that single GSCs detach and move away from the hub after irradiation. The occurrence of single marked germ cells in the differentiation zone declined at later time points after irradiation, suggesting that irradiation-induced GSC detachment from the niche is a transient phenomenon (Fig. 3F).

To identify the fate of detached GSCs post irradiation, we again performed *ex-vivo* live imaging of testes expressing GMA in germ cells under the control of the *nanos* promoter. We added LysoTracker, which selectively labels acidifying cellular compartments, to the live imaging solution to identify dying cells (Zohar-Fux et al., 2022) and imaged each testis for a longer duration (19 h). We never observed a GSC that was still attached to the hub uptake LysoTracker, confirming that GSCs are not dying in the niche. Most of the GSCs that detached from the hub as a single cell moved away from the hub but showed no other detectable changes during the remaining time they were imaged (*n*=6/10 GSCs). A few detached single GSCs later divided (*n*=3/10 GSCs), and one later took up LysoTracker, suggesting that it died after detaching (*n*=1/10 GSCs; Movie 2, Fig. 3I). GSCs that detached as a pair, while still connected to a daughter cell, had similar fates: most had no detectable change, while one divided and one died after detaching (Movies 3, 4, Fig. 3I). Death post detachment was an infrequent event, suggesting that impending death is not the cause of GSC detachment after irradiation. In support of this conclusion, one GSC detached, divided, and then returned to contact the hub (Movie 5). Together, these data reveal that irradiation causes GSCs to detach from the niche rather than die in the niche. Detached GSCs primarily remain in the differentiation zone, occasionally dividing but rarely dying.

### Canonical DNA damage response signaling promotes rapid GSC loss post irradiation

**Fig. 4.**
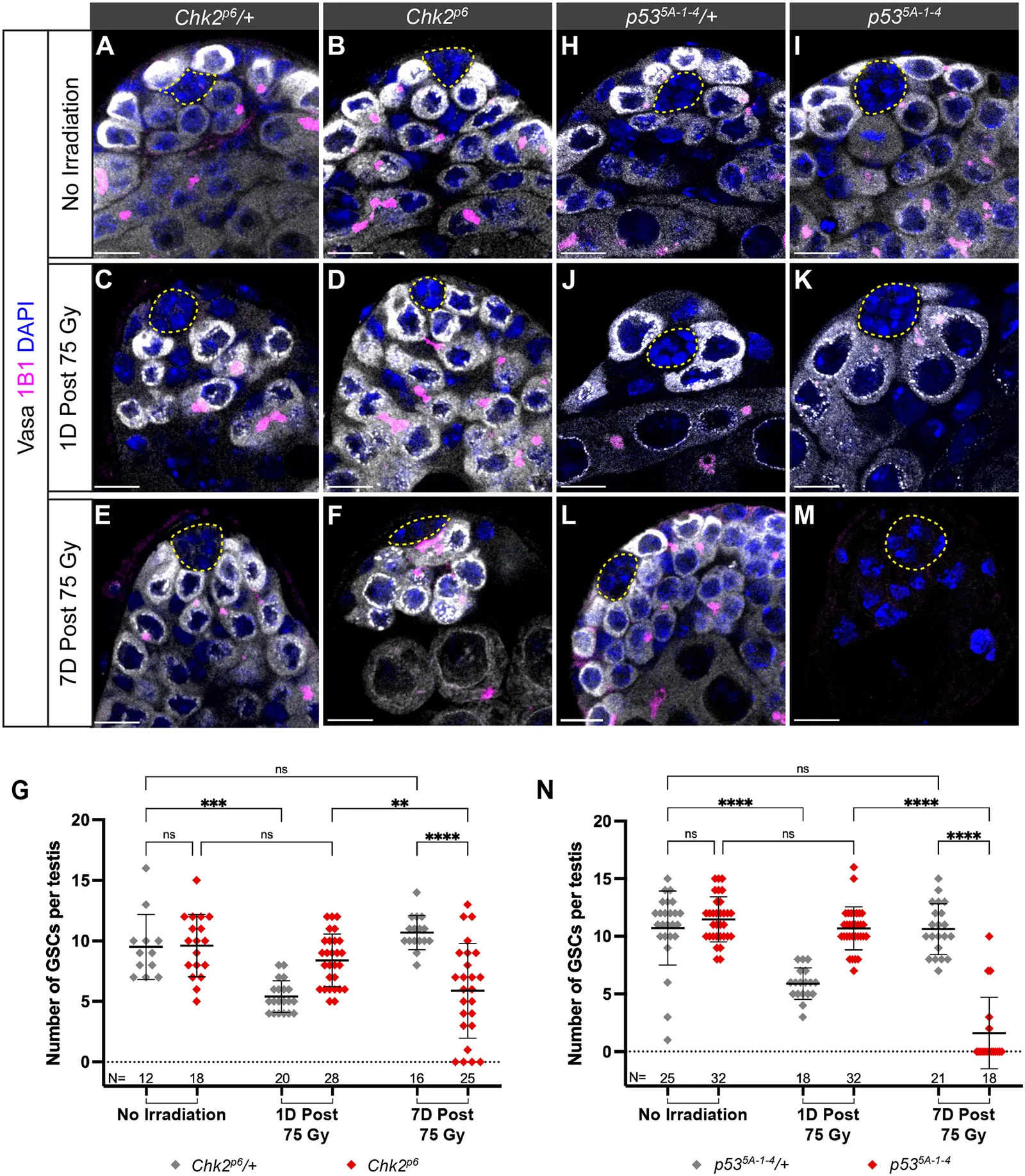
**GSC loss after irradiation requires the canonical DNA damage response pathway members Chk2 and p53.** (A-F,H-M) Confocal sections through the apex of testes immunostained with anti-Vasa (germ cells; white), anti-1B1 (fusomes; magenta) and DAPI (DNA; blue). The hub is outlined in yellow. Homozygous *Chk2* or *p53* mutant or heterozygous control testes are with or without irradiation as indicated. Scale bars: 10 μm. (G,N) Quantification of the data from A-F,H-M showing that heterozygous controls (gray) initially lose but then regain GSCs by 7 days after irradiation. By contrast, in homozygous *Chk2* or *p53* mutant (red) testes initially retain but then lose GSCs by 7 days after irradiation. **P<0.01, ***P<0.001, ****P<0.0001 (ordinary two-way ANOVA with Tukey's multiple comparisons). ns, not significant. Bars indicate mean±s.d. N=number of testes.

We next investigated which pathways mediate irradiation-induced GSC loss. As GSCs sustain high levels of DNA damage after irradiation (Fig. S1), we hypothesized that the canonical DNA damage response machinery could be involved. A key component of this machinery is Chk2, which becomes activated and phosphorylates downstream factors in response to DNA double-strand breaks (Clay and Fox, 2021; Masrouha et al., 2003). To determine whether GSC loss depends on Chk2, we examined testes from homozygous null mutant *Chk2^p6^* males, which completely lack Chk2 but are otherwise viable and fertile (Abdu et al., 2002). Testes from non-irradiated *Chk2^p6^* males were phenotypically indistinguishable from control testes (here, *Chk2^p6^/+* males) (Fig. 4A,B,G). By contrast, 1 day post irradiation, although the number of GSCs fell significantly as expected in control testes, GSCs were retained in *Chk2^p6^* testes (Fig. 4C,D,G). To confirm these results, we examined testes from males carrying *Chk2^p6^* combined with a kinase-dead allele, *Chk2^KD^*, which lacks kinase activity (Ma et al., 2016). Without irradiation, *Chk2^KD^/Chk2^p6^* testes contained slightly fewer GSCs than control *Chk2^KD^/+* males (Fig. S3A,B,G). However, 1 day post irradiation, although GSCs were lost as expected in control testes, they were again retained in testes from *Chk2^KD^/Chk2^p6^* males (Fig. S3C,D,G). These findings suggest that GSC loss after irradiation depends on the kinase activity of Chk2, which is consistent with work in the *Drosophila* ovary, where Chk2 is required for GSC loss following X-irradiation (Ma et al., 2016). We next investigated whether p53 is required for GSC loss after irradiation. p53 is a target of Chk2 and its activity is detectable in germ cells after 40 Gy irradiation (Brodsky et al., 2004; Wylie et al., 2014; Zannini et al., 2014). As with Chk2, homozygous null mutant *p53^5A-1-4^* males are viable and fertile (Xie and Golic, 2004), and we found their testes to be phenotypically indistinguishable from control (*p53^5A-1-4^/+*) testes (Fig. 4H,I,N). One day post irradiation, significant GSC loss was evident in control testes but not in *p53^5A-1-4^* testes (Fig. 4J,K,N). Confirming this result, testes from another mutant combination, p53*^5A-1-4^/p53^11-1B-1^* (Rong et al., 2002), also retained GSCs at 1 day post irradiation while GSCs in control *p53^11-1B-1^*/+ testes were significantly reduced (Fig. S3H-K,N). As GSCs were not lost in testes lacking either Chk2 or p53, we conclude that the canonical DNA damage response machinery is required for the initial loss of GSCs after irradiation.

To determine whether Chk2 and p53 are required cell-autonomously to mediate GSC loss after irradiation, we used the Gal4-UAS system (Brand and Perrimon, 1993) to either knock down expression of Chk2 with *UAS-Chk2-RNAi*, or overexpress a dominant-negative form of p53, *UAS-p53^R155H^* (Ollmann et al., 2000), using a germ cell-specific driver, *nanos-Gal4*; flies carrying the *nanos-Gal4* driver alone were used as controls. Without irradiation, testes from control and germline-specific Chk2 knockdown or p53^R155H^ overexpression flies had similar numbers of GSCs (Fig. S4A-C,J). One day post irradiation, GSC numbers declined in control testes but not in testes with germline-specific Chk2 knockdown or p53^R155H^ overexpression (Fig. S4D-F,J), indicating that the canonical DNA damage response machinery is required cell-autonomously, within GSCs, for their initial loss after irradiation.

To assess the longer-term fate of GSCs lacking Chk2 or p53 after irradiation, we quantified the number of GSCs in *Chk2* and *p53* mutant testes at 7 days post irradiation. In contrast to control testes, which had regained a normal number of GSCs as expected, *Chk2^p6^*, *p53^5A-1-4^* and *p53^5A-1-4^/p53^11-1B-1^* testes now had significantly reduced numbers of GSCs (Fig. 4E-G,L-N, Fig. S3L-N). GSCs were not significantly reduced in *Chk2^KD^/Chk2^p6^* testes (Fig. S3E-G) or in testes with germline-specific Chk2 knockdown or p53 dominant-negative overexpression (Fig. S4G-J) at 7 days post irradiation, although the number of GSCs trended downward in the latter, suggesting that GSCs might be lost more slowly in these genotypes. Taken together, these results suggest that GSCs lacking Chk2 or p53 initially remain in the niche after irradiation but are eventually lost over time, perhaps through alternative pathways.

We speculated GSCs in *Chk2* and *p53* mutant testes could be lost over time because they fail to repair damaged DNA. To test this hypothesis, we immunostained *p53^5A-1-4^* homozygous mutant and control (heterozygous) testes for γH2AvD to identify DNA double-strand breaks. One hour post irradiation, both *p53^5A-1-4^* and control testes showed robust γH2AvD staining throughout the testis apex (Fig. S5A-B″). GSCs in both *p53^5A-1-4^* and control testes continued to show robust γH2AvD staining at 1 day post irradiation; by 7 days post irradiation, however, γH2AvD staining was no longer detectable in control GSCs but remained at high levels in GSCs in *p53^5A-1-4^* testes, indicating that DNA double-strand breaks had not been repaired in GSCs lacking p53 (Fig. S5C-F′). Intriguingly, in both *p53^5A-1-4^* and control testes, γH2AvD staining was reduced in adjacent CySCs by 1 day post irradiation and undetectable by seven days (Fig. S5C-F″). We conclude that GSCs lacking p53 initially remain at the hub but are eventually lost after irradiation because they are unable to repair DNA double-strand breaks. By contrast, these breaks can be repaired in somatic stem cells (CySCs) independently of p53. The decision of GSCs to detach or remain in the niche within 24 h of irradiation is likely a process downstream of p53. The failure of Chk2- or p53-deficient testes to lose their GSCs 1 day post irradiation reveals the essential signaling role the canonical DNA damage response pathway has after genomic insult.

### c-NHEJ is not required for GSC recovery post irradiation

**Fig. 5.**
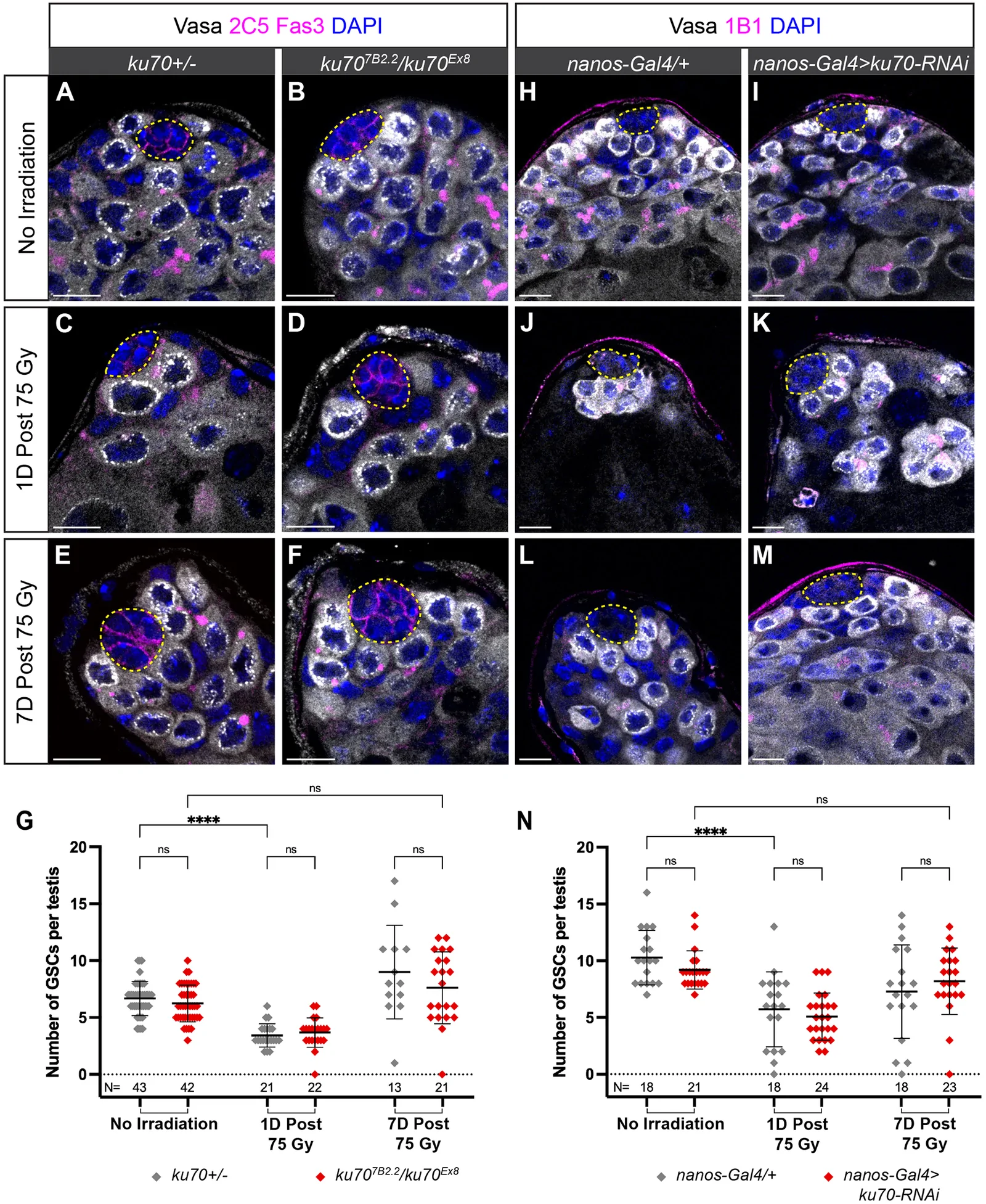
**Ku70, a member of the c-NHEJ pathway, is not required for GSC regeneration after irradiation.** (A-F) Confocal sections through the apex of testes immunostained with anti-Vasa (germ cells; white), anti-2C5 (fusomes; magenta), anti-Fasciclin 3 (hub cells; magenta) and DAPI (DNA; blue). *ku70* null mutant or heterozygous control testes are with or without irradiation as indicated. The hub is outlined in yellow. Scale bars: 10 μm. (G) Quantification of the data from A-F showing that both *ku70* null mutant (red) and heterozygous control (gray) testes initially lose and then regain GSCs by 7 days after irradiation. (H-M) Confocal sections through the apex of testes stained and outlined as in A-F. Control testes (*nanos-Gal4/+*) and testes expressing RNAi against *ku70* in germ cells (*nanos-Gal4>ku70-RNAi*) are with or without irradiation as indicated. Scale bars: 10 μm. (N) Quantification of the data from H-M shows that both control testes (*nanos-Gal4/+*) and testes expressing *ku70-RNAi* in germ cells initially lose and then regain GSCs by 7 days after irradiation. For both quantifications (G,N), ****P<0.0001 (ordinary two-way ANOVA with Tukey's multiple comparisons test). ns, not significant. Bars indicate mean±s.d. N=number of testes.

We next sought to determine whether the surviving GSCs preferentially use a specific DNA double-strand break repair pathway post irradiation. We first investigated the requirement of c-NHEJ using either *ku70* or *lig4* null mutant males, which are viable and fertile but lack these essential components of the c-NHEJ pathway (Johnson-Schlitz et al., 2007). Testes from null mutant *ku70^7B2.2^/ku70^Ex8^* males without irradiation were phenotypically indistinguishable from heterozygous sibling controls (Fig. 5A,B,G). One day post irradiation, both the controls and the *ku70^7B2.2^/ku70^Ex8^* null mutant testes similarly lost GSCs, and then fully recovered their GSCs by 7 days post irradiation (Fig. 5C-G). Consistent with this result, we could no longer detect bright γH2AvD staining at the apex of these testes, suggesting a full resolution of DNA damage foci by 7 days post irradiation in both control and *ku70* null mutant males (Fig. S6A-H). Similarly, in *lig4^5^/Y* null mutant testes (Gorski et al., 2003), GSCs were lost 1 day post irradiation but fully recovered by 7 days later (Fig. S7A-D). These results indicate that neither *ku70* nor *lig4* is required for recovery of GSCs after irradiation. To confirm these results, we knocked down *ku70* expression specifically in germ cells by driving expression of *UAS-ku70-RNAi* with *nanos-Gal4*. Without irradiation, control (*nanos-Gal4* driver only) and knockdown testes had comparable numbers of GSCs (Fig. 5H,I,N). Similarly, both control and knockdown testes lost GSCs 1 day post irradiation and fully regained them by 7 days (Fig. 5J-N). Together, these results indicate that the c-NHEJ pathway is not required for GSC loss or regeneration after irradiation.

### HR is required for GSC recovery post irradiation

**Fig. 6.**
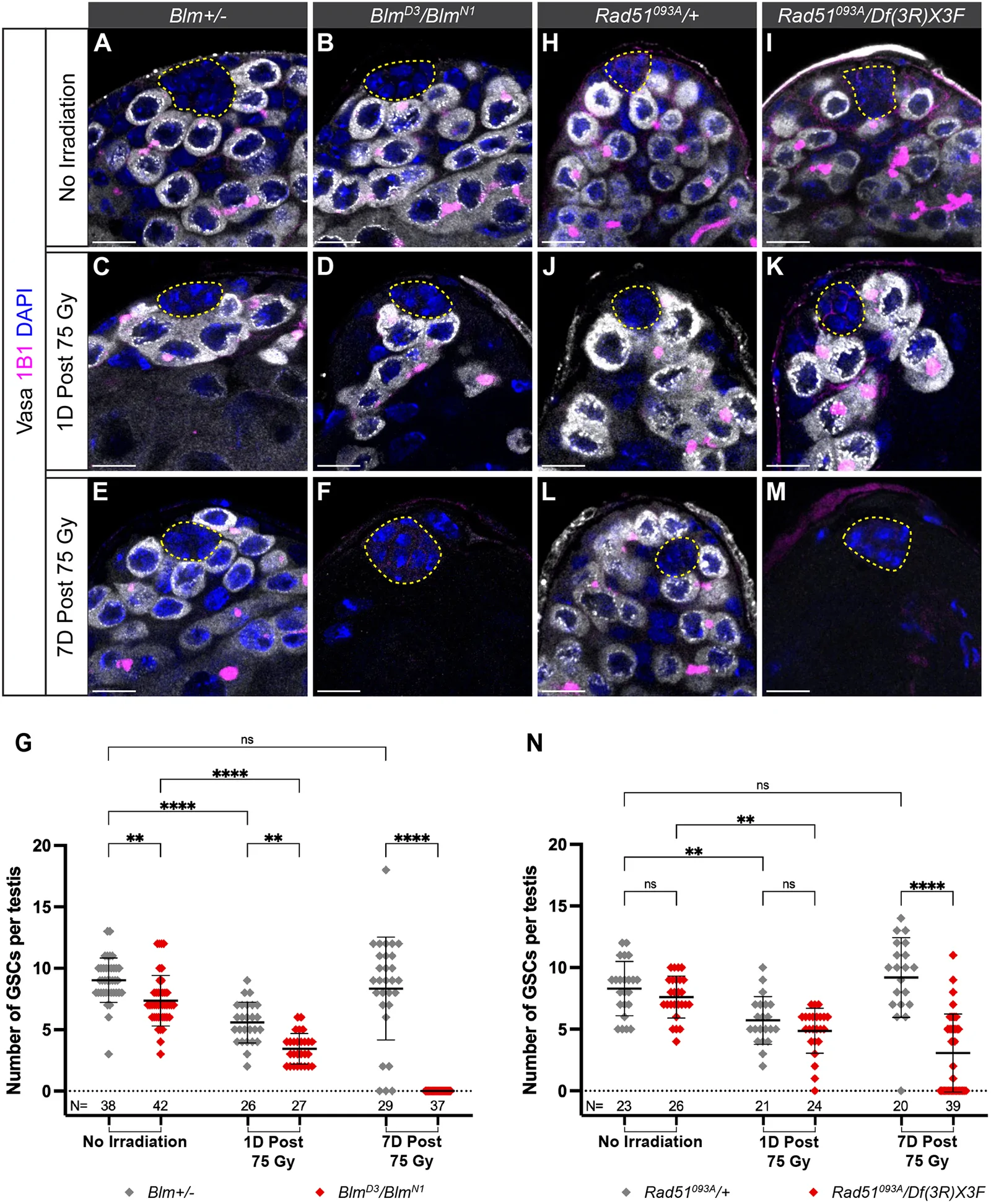
**GSC regeneration after irradiation requires components of the homologous recombination repair pathway.** (A-F,H-M) Confocal sections through the apex of testes immunostained with anti-Vasa (germ cells; white), anti-1B1 (fusomes; magenta) and DAPI (DNA; blue). The hub is outlined in yellow. *Blm* or *Rad51* mutant or heterozygous control testes are with or without irradiation as indicated. Scale bars: 10 μm. (G,N) Quantification of the data from A-F,H-M showing that both *Blm* or *Rad51* mutant testes (red) and heterozygous control testes (gray) initially lose GSCs after irradiation. By 7 days, however, although most control testes regain GSCs, *Blm* or *Rad51* mutant testes do not. **P<0.01, ****P<0.0001 (ordinary two-way ANOVA with Tukey's multiple comparisons). ns, not significant. Bars indicate mean±s.d. N=number of testes.

We next tested the requirement for the HR pathway in the GSCs post irradiation by examining mutant flies lacking *Bloom syndrome helicase* (*Blm*), which is essential for HR repair in *Drosophila* (Johnson-Schlitz and Engels, 2006; Johnson-Schlitz et al., 2007). To knock out *Blm* function, we combined the null mutant *Blm^N1^* with the helicase-dead mutant *Blm^D3^* (Boyd et al., 1981; Kusano et al., 2001; McVey et al., 2007). Without irradiation, the mutant *Blm^D3^/Blm^N1^* testes had reduced numbers of GSCs compared to heterozygous sibling controls (Fig. 6A,B,G). One day after irradiation, the *Blm^D3^/Blm^N1^* and control testes had a similar drop in GSC numbers (Fig. 6C,D,G). Surprisingly, while the GSC numbers of control testes returned to normal 7 days post irradiation, there was no GSC recovery in *Blm^D3^/Blm^N1^* mutant testes (Fig. 6E-G), suggesting that the HR pathway is essential for regeneration of GSCs post irradiation. This result is unexpected because loss of HR has been shown to be compensated for by other DNA double-strand break repair pathways in various tissues across many species (Johnson-Schlitz et al., 2007; Tan et al., 2023). We speculated that GSCs are not recovered post irradiation in *Blm* mutant testes because they are unable to repair DNA double-strand breaks, which we assessed by staining for γH2AvD (Fig. S6I-R′). However, even by 3 days post irradiation, almost all GSCs were lost from the niche, posing a challenge in determining a direct connection between the presence of double-strand breaks and the absence of the HR pathway.

To further test the requirement for the HR pathway in GSC regeneration post irradiation, we next looked at mutants for Rad51, which encodes another essential component of the HR repair pathway in *Drosophila* (Johnson-Schlitz et al., 2007; Sekelsky, 2017). We combined the null mutant *Rad51^093A^* (Staeva-Vieira et al., 2003) with *Df(3R)X3F*, a chromosome carrying a deletion that completely removes the *Rad51* gene. Without irradiation, *Rad51^093A^/Df(3R)X3F* testes had similar numbers of GSCs compared to heterozygous control testes (Fig. 6H,I,N). As with *Blm* mutants, GSC numbers per testis were reduced in both the *Rad51^093A^/Df(3R)X3F* and control testes 1 day after irradiation (Fig. 6J,K,N). By contrast, 7 days post irradiation, the number of GSCs remained significantly low in *Rad51^093A^/Df(3R)X3F* null testes*,* but had fully recovered in control testes (Fig. 6L-N). These observations confirm that the HR pathway is essential for the successful regeneration of GSCs after DNA damage-induced loss.

To determine whether GSC loss and regeneration post irradiation requires the HR pathway cell-autonomously, we knocked down expression of Rad51 specifically in germ cells again using the *nanos-Gal4* driver together with *UAS-Rad51-RNAi*. Without irradiation, Rad51 knockdown testes were phenotypically indistinguishable from driver controls (Fig. S8A,B,G). One day post irradiation, both the *Rad51* knockdown and control testes had lost GSCs (Fig. S8C,D,G). By contrast, 7 days post irradiation, control testes had regained a full complement of GSCs, while the GSCs lacking *Rad51* in early germ cells largely failed to recover (Fig. S8E-G). Taken together, these data indicate that GSCs unable to use HR cannot be recovered post irradiation. Our results highlight the role of HR in GSC regeneration after significant DNA damage-induced GSC loss in the *Drosophila* testis and strongly suggest that, unlike most cells, which can use repair pathways redundantly, adult male GSCs preferentially use HR, and not NHEJ, to repair irradiation-induced DNA double-strand breaks.

## DISCUSSION

This study supports a model wherein testes in adult male *Drosophila* lose GSCs in a Chk2- and p53-dependent manner within a day of irradiation at 75 Gy. The surviving GSCs either complete DNA double-strand break repair and remain in the niche or detach from the niche to die or divide. GSCs repair DNA double-strand breaks using the HR pathway rather than NHEJ pathway, unlike most other cells, which can use these pathways redundantly. Within a week post irradiation, niches in most testes fully regain their GSCs.

### Irradiation induces cellular behavior change in testis GSCs

Different stem cell populations vary in their resistance and cellular response to irradiation depending on the tissue type (Blanpain et al., 2011). The GSCs of male *Drosophila* are more radioresistant relative to their differentiated progeny, in part due to niche signals that promote anti-apoptotic factors in the stem cells (Hasan et al., 2015). However, the cellular dynamics of GSCs post irradiation have never been directly observed. Here, whole-mount live imaging of the testis post irradiation revealed that approximately a quarter of the GSCs detach and move away from the hub as single cells, which is a cellular behavior not seen in non-irradiated control testes (Sheng and Matunis, 2011). This detachment could result from altered niche signaling causing a change in cell adhesion or migration, as has been reported in some mammalian cell types post irradiation (Ma et al., 2025; O'Sullivan et al., 2020). Since our current live-imaging approach does not extend past 24 h, we are not able to determine whether some detached GSCs die at later time points after irradiation, or if re-associating GSCs remain at the niche for as long as GSCs that were present before irradiation.

The Janus kinase–Signal transducer and activator of transcription (JAK-STAT) signaling pathway, which mediates GSC adherence to the hub via E-cadherin in the *Drosophila* testis (de Cuevas and Matunis, 2011), is a candidate regulator of GSC attachment post irradiation. Why some GSCs remain in the niche and others detach is not known but could be stochastic or reflect variation in DNA repair levels in the GSCs. Future insight could come from investigating the distribution of E-cadherin post irradiation or adapting laser microirradiation (Kim et al., 2019) to follow the fates of individually irradiated GSCs and their non-irradiated neighbors.

### DNA damage induces Chk2- and p53-dependent loss and regeneration of GSCs

The activation of Chk2 kinase upon DNA damage is a conserved process across many model organisms that regulates DNA damage repair, cell cycle arrest, and apoptosis (Clay and Fox, 2021). In *Drosophila*, Chk2 activity has been well characterized in the embryo (Masrouha et al., 2003), larval wing disc (Wells and Johnston, 2012) and adult ovary (Ma et al., 2016). In this study, we highlight the role of Chk2 in adult *Drosophila* testes to modulate GSC loss in response to irradiation. A similar cell loss phenotype has been observed after Chk2 depletion in other systems. For example, mouse thymocytes and neurons undergo Chk2-dependent apoptosis in response to irradiation (Takai et al., 2002). Similarly, the GSCs of the *Drosophila* ovary also undergo a Chk2-dependent loss post irradiation (Ma et al., 2016).

Chk2 mediates apoptosis in response to radiation via p53 in *Drosophila* larval wing discs (Xu et al., 2001) and embryos (Brodsky et al., 2004). In the *Drosophila* ovary and testis, *p53* activity is detected in the GSCs and immediate daughters after irradiation (Wylie et al., 2014). However, the requirement of p53 for stem cell survival after irradiation differs between the two tissues. In the ovary, irradiation induces Chk2-dependent, p53-independent GSC loss, resulting in the accumulation of single cystoblast-like cells (Ma et al., 2016). We observed a similar irradiation-induced loss of GSCs in the testis; however, unlike in the ovary, both p53 and Chk2 are required for this loss.

Activation of p53 can induce many different responses, including cell cycle arrest, DNA damage repair, and cell death if the damage is too severe (Liu et al., 2024). Here, we show that p53 mediates a response whereby a portion of GSCs are lost in a Chk2- and p53-dependent manner immediately after irradiation. A similar response has been seen in the developing central nervous system of mice (Herzog et al., 1998); in the *Drosophila* embryo, where p53 activation induces cell death (Brodsky et al., 2004); and in the *Drosophila* ovary where p53 is necessary and sufficient for irradiation-induced apoptosis of germline and somatic cells (Chakravarti et al., 2022). Thus, p53 plays a role in both the ovary and the testis in maintaining GSCs in the stem cell niche as the tissue recovers from damage.

### GSCs require HR and not NHEJ to repair DNA double-strand breaks

The primary double-strand break repair pathway a cell uses may reflect the needs of that cell type. Since HR is non-mutagenic while NHEJ is error prone, it could be advantageous for GSCs to preferentially use the HR pathway to prevent mutations from being passed to subsequent generations. Consistent with our finding that male GSCs specifically require the HR pathway, repair reporter studies have shown that pre-meiotic germ cells (GSCs and spermatogonia) lacking NHEJ compensate by using HR, but loss of HR is not compensated for by NHEJ (Johnson-Schlitz et al., 2007). However, repair reporters also indicate that pre-meiotic germ cells in young males can use NHEJ and HR when both pathways are available (Preston et al., 2006). This potential difference with our findings is likely due to the cells surveyed (GSCs alone versus all pre-meiotic germ cells), which could have distinct repair pathway requirements. Overall, our work poses an interesting question as to why the GSCs in the testis fail to use NHEJ upon DNA damage when HR is unavailable. The underlying mechanisms that ensure repair occurs by HR in GSCs are an important focus for future work.

### Implications for radioresistant cancer stem cells

Studying the diversity of stem cells' response to radiation is essential to understanding how stem cells preserve the genome and regenerate tissues. Humans are routinely exposed to radiation throughout life from various sources, such as cosmic rays, medical X-rays, airport security, naturally occurring radon gas, and cancer treatments (Cannan and Pederson, 2016). Many tumors contain a population of cancer stem cells that are not only radioresistant, but expansion of which can be stimulated by irradiation (Chu et al., 2024; Ghisolfi et al., 2012). The ability of the cancer stem cells to repair DNA damage contributes to the recurrence of the tumor. Certain cancer stem cell populations, such as glioblastoma and glioma, prefer HR-mediated DNA damage repair (Schulz et al., 2019), as we have found in *Drosophila* male GSCs. Thus, understanding the role of DNA damage response pathways in a variety of stem cell niches could inform future treatment strategies in diseases like cancer.

### Study limitations

One limitation of this study is the inability to distinguish the progeny of re-associating GSCs, which arise post irradiation, from the progeny of GSCs that were present before irradiation. Therefore, we could not determine whether re-associating GSCs go on to produce functional sperm.

## MATERIALS AND METHODS

### Fly stocks and culture

Flies were raised on standard molasses media with sprinkling of dry yeast at 25°C. Unless otherwise specified, control males were *y^1^ w^1118^*. The following stocks were used: *UASp-p35-FLAG* (a gift from Ting Xie; Ma et al., 2016), *mnk^p6^* (a gift from Zhao Zhang; Ma et al., 2016), *nanos-Gal4* (a gift from Erica Selva; Van Doren et al., 1998), *nanos-Gal4* (a gift from Mark Van Doren, Johns Hopkins University - Krieger School of Arts and Sciences, MD, USA), *SpnA^093A^/TM3* (Staeva-Vieira et al., 2003), *y w;ku70^7B2.2^/TM6B Tb Hu, y w;ku70^Ex8^/TM6B Tb Hu* (gifts from Jan LaRocque, Georgetown University, DC, USA; Johnson-Schlitz et al., 2007) and *HS-FLP/Y; Tubulin>STOP>Gal4, UAS GFP* (a gift from Ben Ohlstein, UT Southwestern Medical Center, TX, USA). Other stocks were sourced from the Bloomington *Drosophila* Stock Center (BDSC): *lok^KD^/Cyo* (BDSC, 605592), *UAS-Rad51-RNAi* (BDSC, 38898), *UAS-Chk2-RNAi* (BDSC, 35152) and *UAS-ku70-RNAi* (BDSC, 61942). *UAS-p53^R155H^* (BDSC, 8419) was used to reduce functional p53. *Df(3R)X3F/MKRS* (BDSC, 2352) flies were crossed to *SpnA^093A^/TM3* to obtain flies completely lacking Rad51. *SpnA^093A^/TM3* flies were used as heterozygous controls as described by Staeva-Vieira et al. (2003). *p53^5A-1-4^* (BDSC, 6815) and *p53^11-1B-1^* (BDSC, 6816) were used to study flies lacking p53. *p53^5A-1-4^* and *p53^11-1B-1^* were crossed to *y^1^ w^1118^* flies to obtain heterozygous controls. *Blm^D3^/TM3* (BDSC, 8656) was crossed to *Blm^N1^/TM3* (BDSC, 28878) to obtain *Blm* null flies; *Blm^D3^/TM3* (BDSC, 8656) flies of the same age were used as heterozygous controls. Flies null for *ligase 4* (*lig4^5^*) (BDSC, 8519) were also used. *y w;ku70^Ex8^/TM6B Tb Hu* were crossed to *y w;ku70^7B2.2^/TM6B Tb Hu* to obtain trans heterozygotes to obtain *ku70* null flies; siblings were used as controls. For *ex vivo* live imaging, *nanos::Moe-GFP;His-RFP/TM3,Ser* flies were created from *nanos::Moe-GFP* (a gift from Ruth Lehmann; Sano et al., 2005) and *His2Av-RFP* (BDSC, 6598), or *nanos-Gal4* crossed to *UAS-GMA* (a gift from Dan Kiehart, Duke University, NC, USA; Bloor and Kiehart, 2001; Dutta et al., 2002; Table S1). We used FlyBase (release FB2025_05) to find information on phenotypes, function, stocks and gene expression (Öztürk-Çolak et al., 2024).

### Transgene expression in GSCs

RNAi expression for knockdown of *ku70*, *Chk2* and *Rad51*, as well as overexpression of *p53^R155H^*, was achieved by crossing virgin *nanos-Gal4* females and males containing the *UAS-RNAi* or *UAS-p53^R155H^* transgene, set at 29°C. Adult male progeny were collected and kept at 29°C for the indicated amount of time before their testes were dissected and fixed.

### Detection of apoptosis and cell death

Staining for apoptosis was carried out using the Millipore ApopTag kit (S7165) following the manufacturer's instruction and the protocol outlined by Hasan et al. (2015). For live imaging of cell death, LysoTracker (Thermo Fisher Scientific, DND-99, L7528) was added to standard live imaging solution for a final concentration of 0.03 μM and allowed to incubate for 1 h prior to the start of the live imaging (modified from Kanaan et al., 2023).

### Quantification of dead cells

Dead cells were quantified by counting TUNEL-positive spots in control and irradiated testes. TUNEL-positive spots in Vasa-positive cells in direct contact with the hub were identified as dead GSCs. TUNEL-positive spots in Vasa-positive cells more than one nucleus diameter away from the hub were identified as dead differentiating germ cells. TUNEL-positive spots in Vasa-positive cells that were not directly contacting the hub, but within one nucleus diameter were identified as dead gonialblasts.

### Quantification of GSCs

GSCs were quantified by manually counting Vasa-positive, 1B1-positive cells directly contacting the hub using the Cell Counter plugin in Fiji (Schindelin et al., 2012).

### Mosaic analysis

Males 0-4 days old of the genotype *HS-FLP/Y; Tubulin>STOP>Gal4, UAS GFP* were heat shocked three times for 40 min each time at 37°C with intervals of 30 min at 25°C to induce clones. Flies were maintained at 29°C for 2 days after clone induction, then irradiated with 75 Gy. After irradiation, flies were maintained at 29°C and dissected 8 h, 1 day, 2 days, 3 days and 7 days after irradiation exposure. Clones were counted as described in the ‘GSCs detach and are displaced from the niche post irradiation’ section.

### Testis immunohistochemistry

Testes were dissected, fixed and immunostained following the protocol described by Matunis et al. (1997). The following primary antibodies were used: rabbit anti-Vasa (1:200; Santa Cruz Biotechnology), rat anti-Vasa (1:50; Developmental Studies Hybridoma Bank), mouse anti-Fasciclin III (1:50; Developmental Studies Hybridoma Bank), mouse anti-Hts (1B1) (1:50; Developmental Studies Hybridoma Bank), mouse anti-Hts (2C5) [1:50; a third unpublished monoclonal raised against the same Hts antigen as antibodies published by Zaccai and Lipshitz (1996); gift from Howard Lipshitz, University of Toronto, Canada], guinea pig anti-Zfh-1 (1:500), chicken anti-GFP (1:10,000; Abcam), mouse anti-FLAG (1:500; Sigma-Aldrich) and rabbit anti-γH2AvD (1:1000; Rockland Immunochemicals). Nuclei were counterstained with 1 μg/ml 4′-6-diamidino-2-phenylindole (DAPI) (Roche Molecular Biochemical). Alexa Fluor-conjugated secondary IgG (H+L) antibodies (Thermo Fisher Scientific) were used at 1:200, 1:100 or 1:66. Catalog numbers and other details regarding primary and secondary antibodies can be found in Table S1. Stained testes were mounted in VECTASHIELD mounting medium (Vector Laboratories) and imaged as described in the ‘Image acquisition’ section.

### Ionizing radiation

Young (0-4 days old) adult male flies were irradiated using a Gammacell 3000 Elan irradiator with Cesium 137 (Best Theratronics) as the radiation source. Flies were treated with 50 Gy, 75 Gy or 100 Gy.

### Image acquisition

Fixed images were acquired using a Zeiss LSM 5 PASCAL microscope, a Zeiss LSM 700 microscope or a Zeiss LSM 800 microscope in the linear range for intensity for all channels. Testes with γH2AvD staining were imaged at the same gain within each experiment. For live imaging, young (0-4 days old) males were irradiated, and control males were dissected immediately after radiation exposure. Testes were isolated, cultured and live imaged as described by Greenspan and Matunis (2023) using a Zeiss LSM 900 confocal microscope (Greenspan and Matunis, 2023). Live imaging solution materials are listed in Table S1. Imaging began 3 h after irradiation exposure. Frames were captured at 25-min intervals. Movies were analyzed manually using the Spots feature in Imaris and then annotated in Fiji.

### Statistical analysis

All statistical tests were performed using Prism version 10 (GraphPad Software).

## Supplementary Material



10.1242/develop.205514_sup1 Supplementary information
