# Supplementary material for: Homologous recombination is essential for DNA damage-induced regeneration of Drosophila testis germline stem cells
Source: Development. 2026 Aug 13;153(15):dev205514. doi: 10.1242/dev.205514 (PMC13535101; doi:10.1242/dev.205514)
Supplement: Supplementary file 1 [file develop-153-205514-s1.pdf]

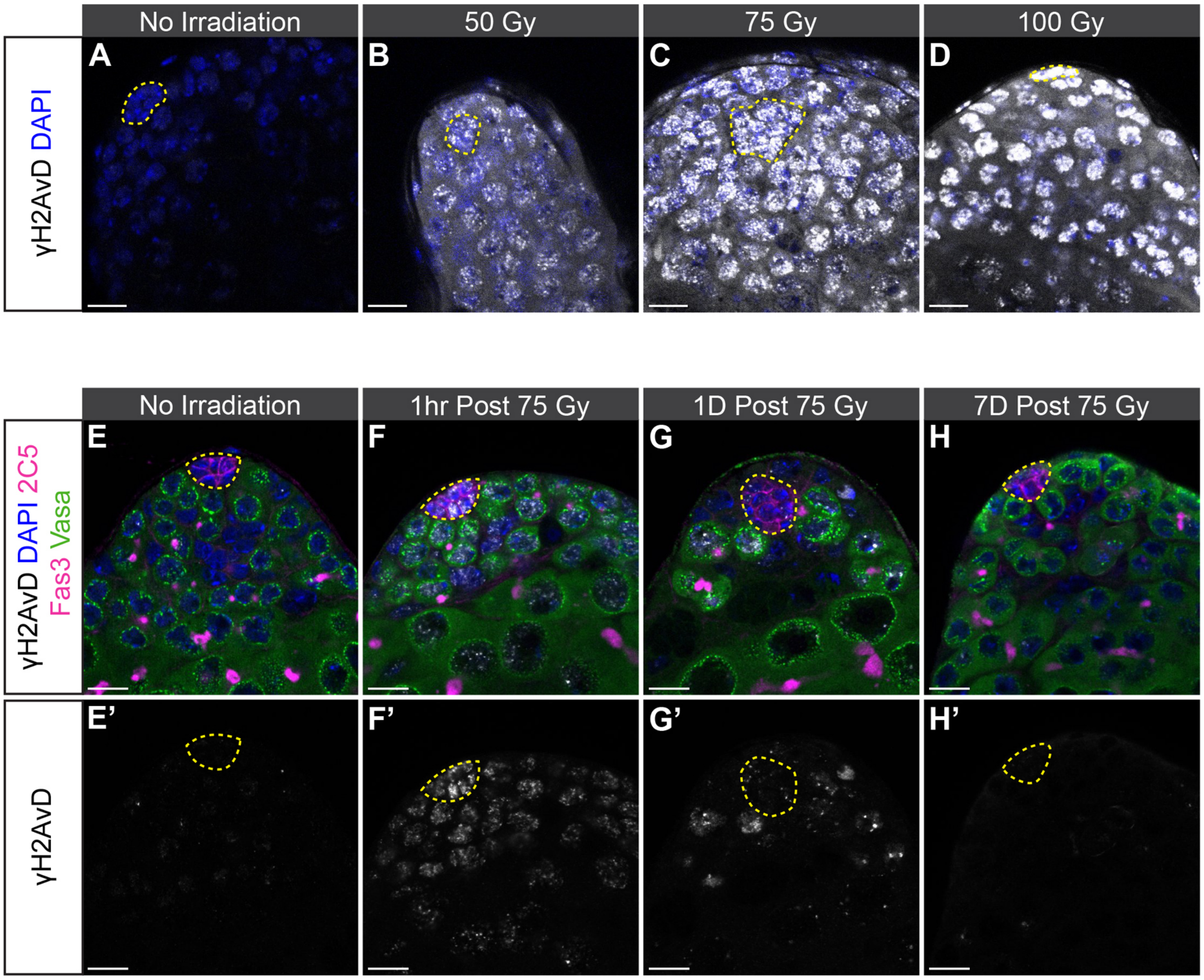

Fig. S1. γH2AvD foci increase with γ-radiation dose in all cells in the testis apex and resolve by 7 days post 75 Gy of irradiation.

(A-D) Confocal sections through the apex of testes immuno-stained with anti-γH2AvD (white) and DAPI 1 hour post irradiation. γH2AvD levels in all cells of the testis apex increase with radiation dose. The hub is outlined in yellow. Scale bar is 10 μm.
(E-H) Confocal sections through the apex of testes immuno-stained with anti-γH2AvD (white), DAPI (blue), anti-2C5 (magenta), anti-Fasciclin 3 (magenta), and anti-Vasa (green).
(E'-H') γH2AvD alone. γH2AvD levels persist from 1 hour to 1 day post irradiation but resolve by 7 days. Scale bar is 10 μm.

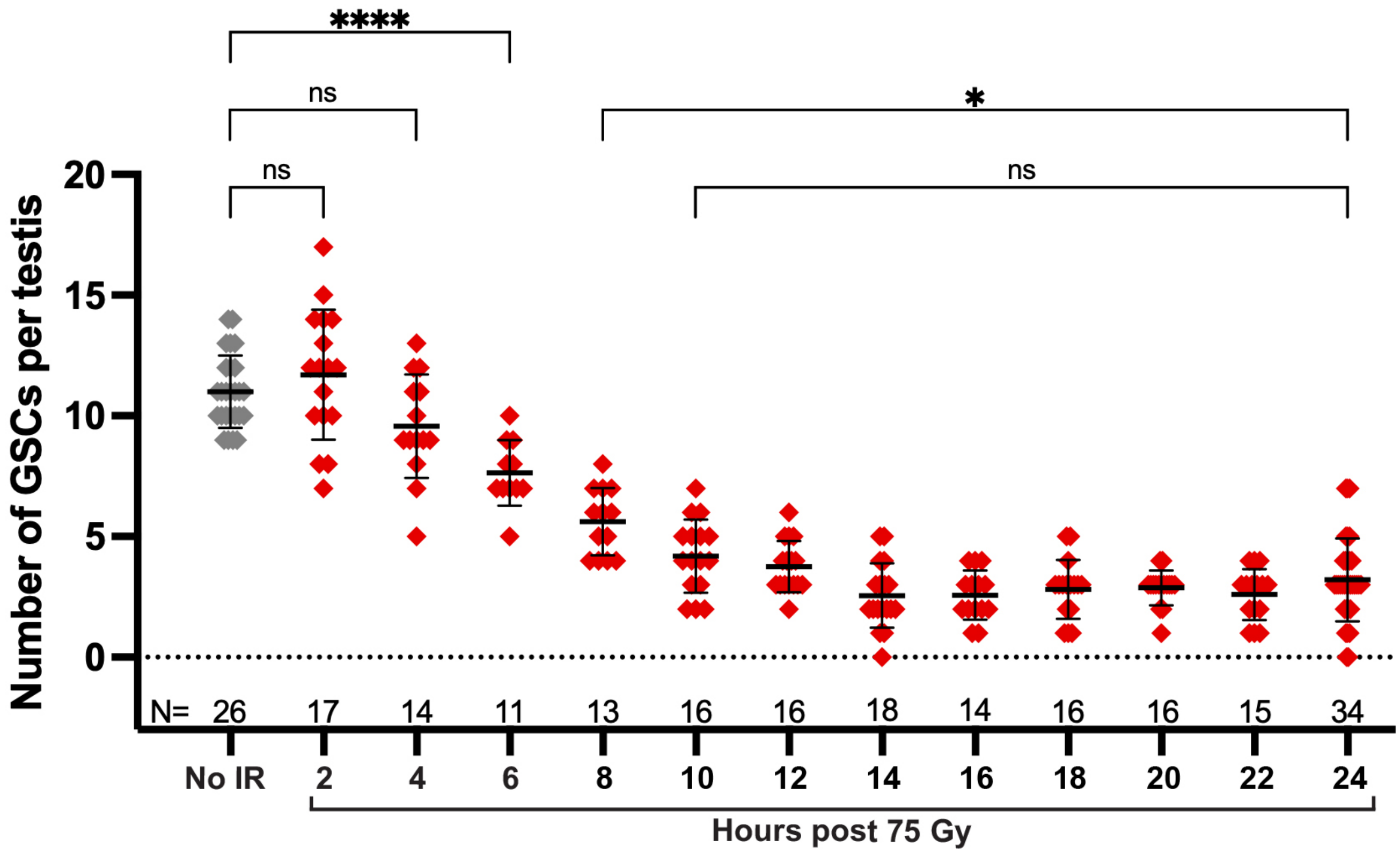

Fig. S2. Significant GSC loss occurs six hours post irradiation, and maximum loss occurs ten hours after irradiation.

Quantification of GSCs per testis in wild-type males post exposure to 75 Gy of irradiation every 2 hours from 2-24 hours. There is a significant reduction in GSC count six hours post irradiation when compared to non-irradiated controls. There is no significant difference in GSC count between 10 – 24 hours post irradiation. * $p < 0.05$, ****$p < 0.0001$, ordinary two-way ANOVA with Tukey's multiple comparisons test. Bars indicate mean and S.D. N = number of testes.

Development • Supplementary information

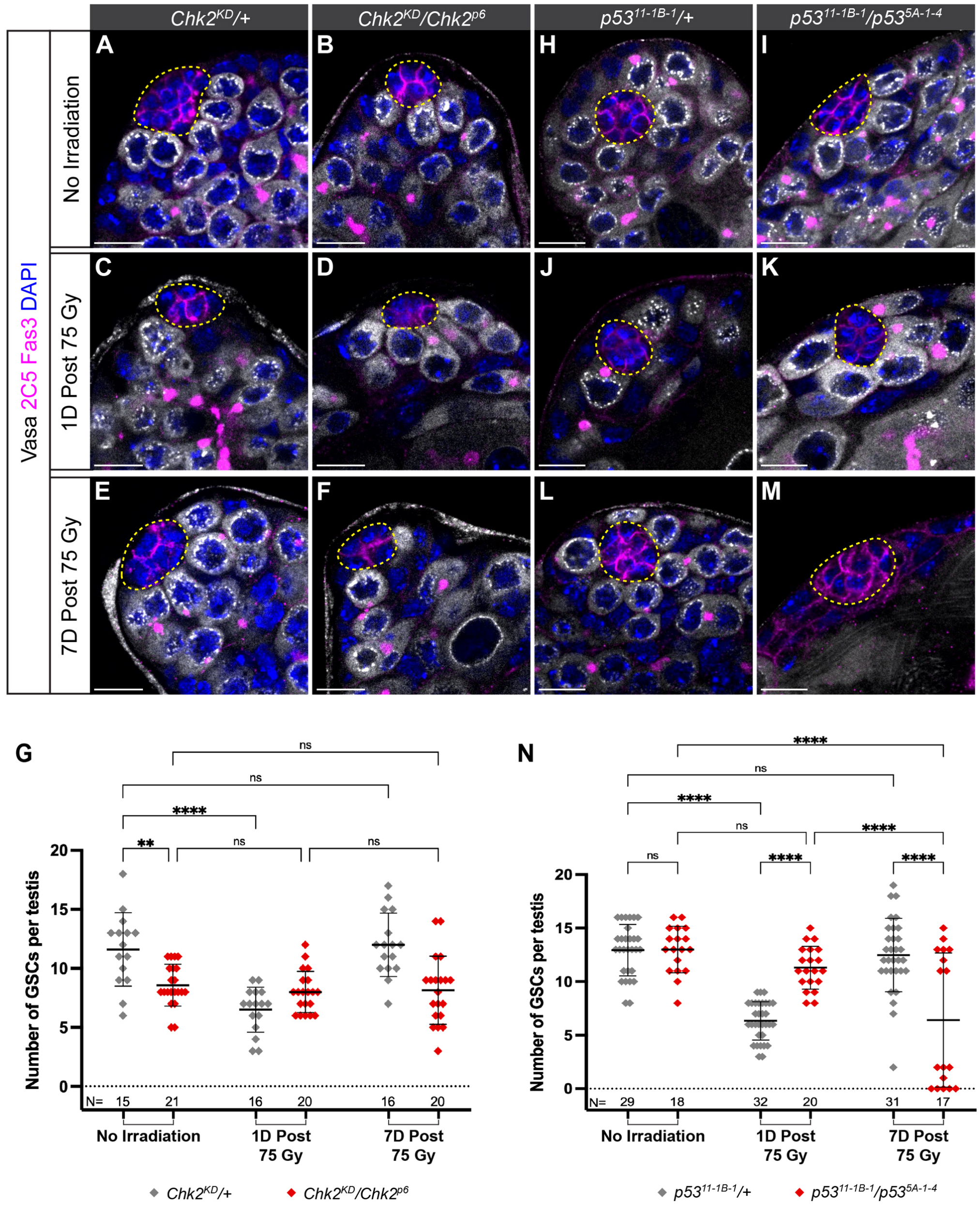

Fig. S3. Additional Chk2 and p53 mutant alleles confirm that GSC loss after irradiation requires the canonical DNA damage response pathway.

(A-F, H-M) Confocal sections through the apex of testes immuno-stained with anti-Vasa (germ cells, white), anti-2C5 (fusomes, magenta), anti-Fasciclin 3 (hub cells, magenta), and DAPI (DNA, blue). The hub is outlined in yellow. Trans heterozygous *Chk2* or *p53* mutant or heterozygous control testes are with or without irradiation as indicated. Scale bar is 10 µm.

(G, N) Quantification of the data from (A-F, H-M) shows that heterozygous controls (gray) initially lose but then regain GSCs by 7 days after irradiation. By contrast, in *Chk2* or *p53* mutants (red), testes initially retain GSCs one day post irradiation. Chk2 mutants maintain GSC numbers through 7 days (G), while p53 mutants lose GSCs by 7 days after irradiation (N). ** $p<0.01$, **** $p<0.0001$, ordinary two-way ANOVA with Tukey's multiple comparisons. Bars indicate mean and S.D. N = number of testes.

Development • Supplementary information

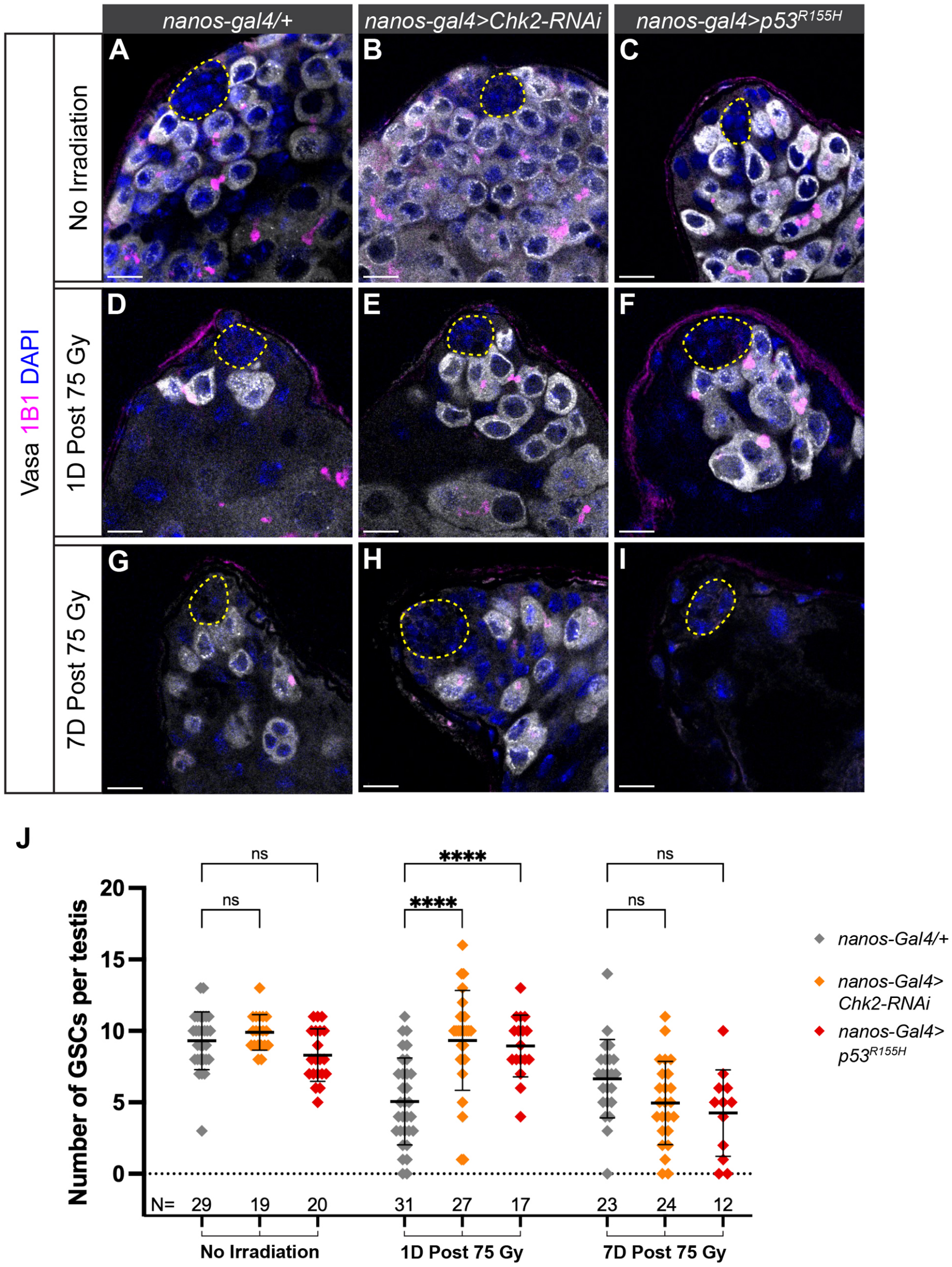

Fig. S4. There is a cell-autonomous requirement of Chk2 and p53 in the germline for DNA damage-induced GSC loss.

(A-I) Confocal sections through the apex of testes immuno-stained with anti-Vasa (marks germ cells, white), anti-1B1 (fusome, magenta) and DAPI (DNA, blue). Hub is outlined in yellow. Driver only control (*nanos-Gal4/+*), *nanos-Gal4>Chk2-RNAi*, or *nanos-Gal4>p53^{R155H}* testes are with or without irradiation as indicated. Scale bar is 10 µm.

(J) Quantification of the data from (A-I) shows that significantly fewer GCSs are observed in irradiated controls one day after irradiation compared to testes expressing *Chk2-RNAi* or *p53^{R155H}* in the germline. GSCs expressing *Chk2-RNAi* or overexpressing *p53^{R155H}* are not significantly different compared to controls but are trending lower. ****p<0.0001, ordinary two-way ANOVA with Tukey's multiple comparisons test. In all graphs, bars indicate mean and S.D. N = number of testes.

Development • Supplementary information

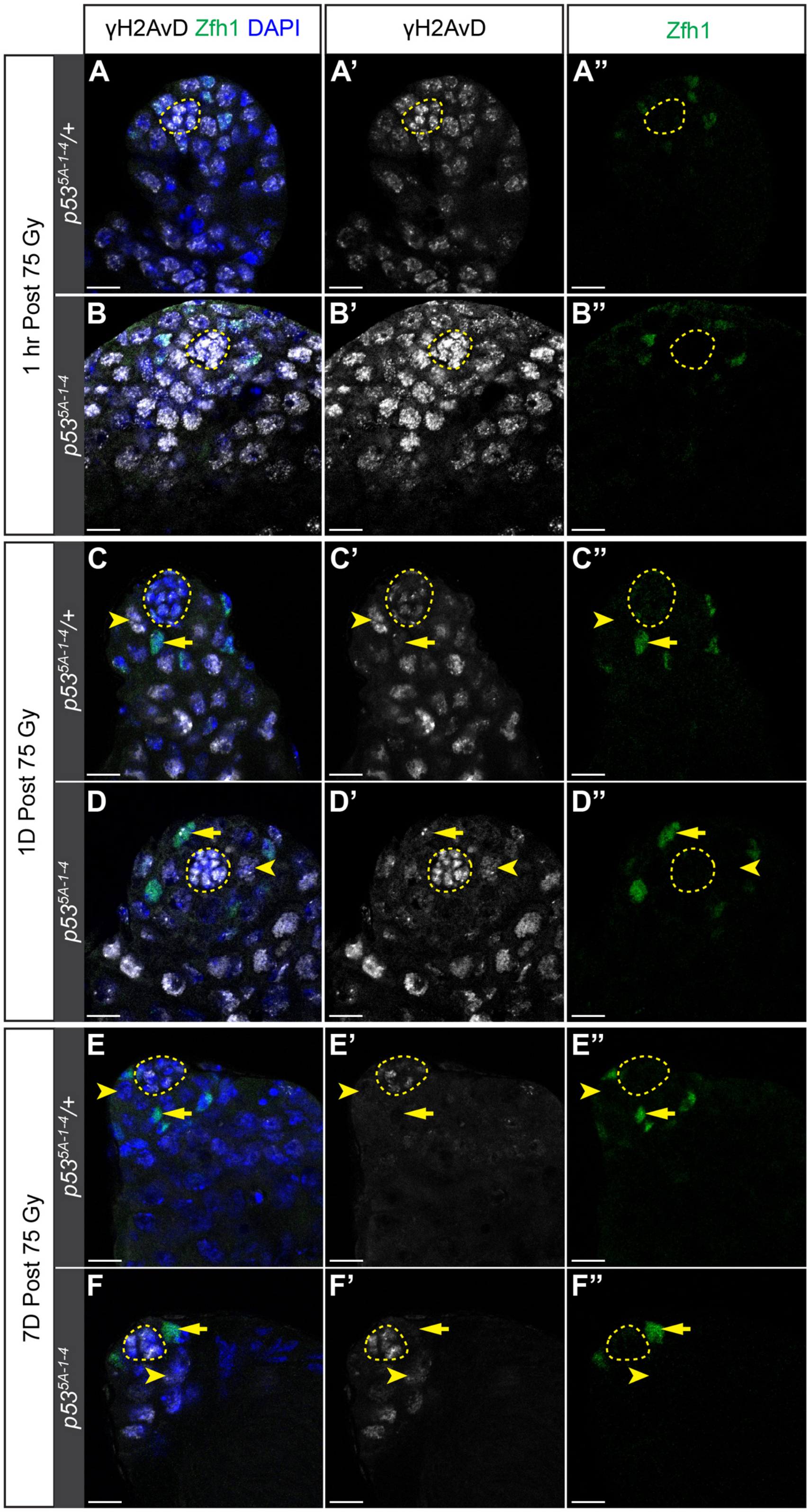
γH2AvD Zfh1 DAPI
γH2AvD
Zfh1
1 hr Post 75 Gy
1D Post 75 Gy
7D Post 75 Gy
p53^5A-1-4/+
p53^5A-1-4
A
A'
A''
B
B'
B''
C
C'
C''
D
D'
D''
E
E'
E''
F
F'
F''

Fig. S5. DNA double strand break repair in GSCs requires p53.
(A-F) Confocal sections through the apex of testes immuno-stained with anti-γH2AvD (white), anti-Zfh1 (CySCs and immediate daughters, green), and DAPI (DNA, blue). The hub is outlined in yellow. Homozygous *p53* mutant or heterozygous control testes are with or without irradiation at 1-hour, 1 day, and 7 days as indicated.

(A-B) 1-hour post irradiation, increased γH2AvD levels are visible in all cells in mutant and control testes.

(C-D) 1 day post irradiation, γH2AvD foci persist in the hub, Zfh-1 positive somatic cells (yellow arrows), and Zfh1 negative GSCs (yellow arrowheads) in mutant and control testes.

(E) 7 days post irradiation, γH2AvD foci decrease in Zfh-1 positive somatic cells (yellow arrows), and Zfh1 negative GSCs (yellow arrowheads), but persist in hub cells in control testes.

(F) *p53* mutant testes retain increased γH2AvD levels in hub and GSCs (yellow arrowhead) but is primarily diminished in CySCs (yellow arrows) in mutant testes.

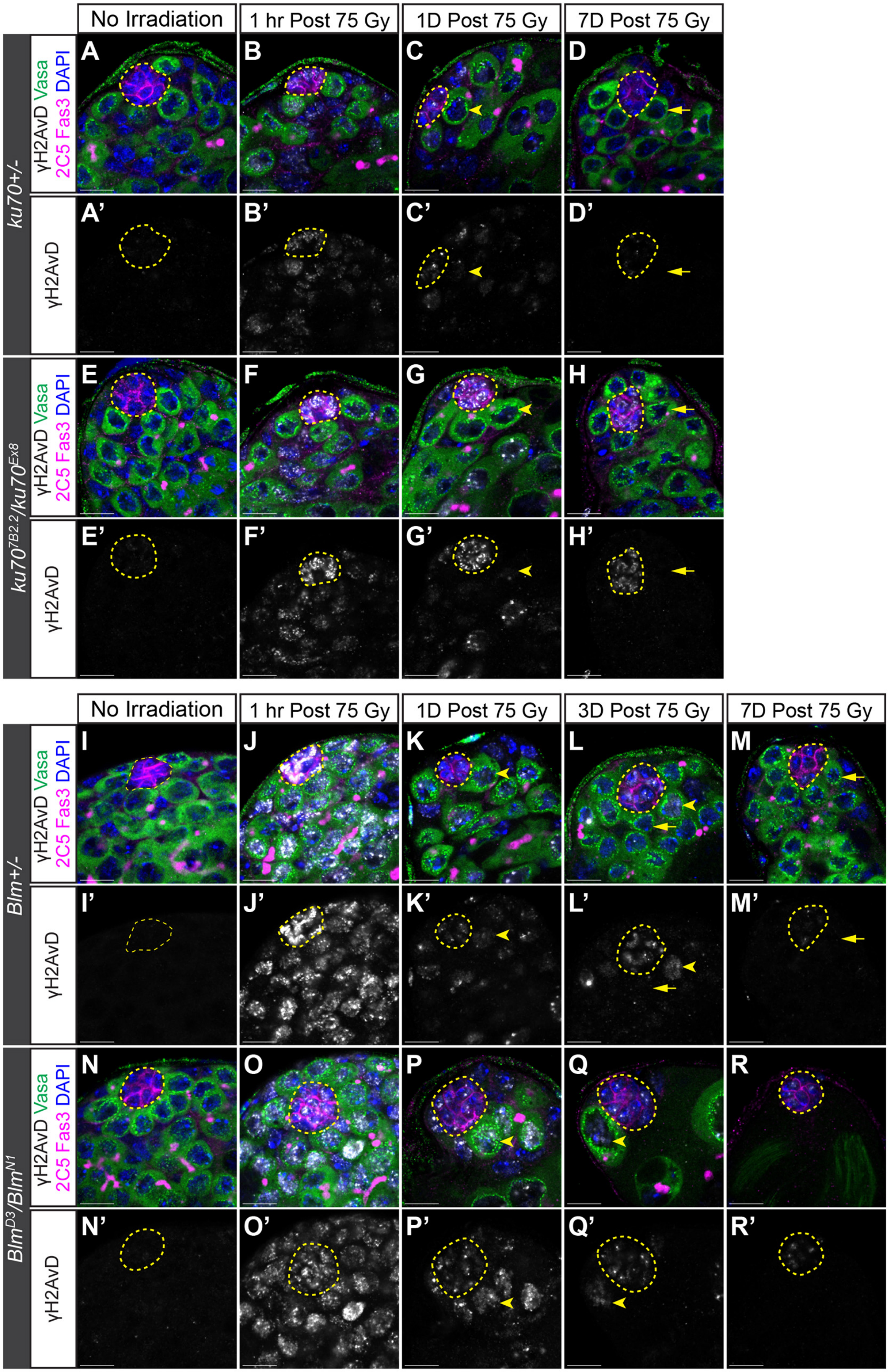

No Irradiation
1 hr Post 75 Gy
1D Post 75 Gy
7D Post 75 Gy
3D Post 75 Gy
ku70+/-
ku70^7B2.2/ku70^Ex8
Blm+/-
Blm^D3/Blm^N1
γH2AvD Vasa 2C5 Fas3 DAPI
γH2AvD
A B C D A' B' C' D' E F G H E' F' G' H' I J K L M I' J' K' L' M' N O P Q R N' O' P' Q' R'

Fig. S6. DNA double strand break repair in GSCs occurs independently of Ku70
(A-R) Confocal sections through the apex of testes immuno-stained with anti-γH2AvD (white), anti-Vasa (germ cells, green), anti-2C5 (fusome, magenta), anti-Fasciclin 3 (hub cells, magenta) and DAPI (DNA, blue). The hub is outlined in yellow.

(A-H) Trans heterozygous *Ku70* mutant or heterozygous control testes are with or without irradiation at 1-hour, 1 day, and 7 days as indicated.

(I-R) Trans heterozygous *Blm* mutant or heterozygous control testes are with or without irradiation at 1-hour, 1 day, 3 days, and 7 days as indicated

(A, E, I, J) Without irradiation, testes show no γH2AvD signal in any cell type.

(B, F, J, O) one hour post irradiation, increased γH2AvD signal is visible in all cells in mutant and control testes.

(C, G) 1 day post irradiation, γH2AvD is decreased in the GSCs in both *Ku70* mutant and control testes (example GSC, yellow arrowhead). The hub of *Ku70* null mutant retains high γH2AvD foci.

(D, H) 7 days post irradiation, γH2AvD signal is diminished throughout *Ku70* mutant and control testes (example GSC, yellow arrow), however the hub of *Ku70* null mutants (H) retains γH2AvD foci.

(K, P) 1 day post irradiation, γH2AvD signal is decreased throughout the testis of control testes (example GSC, yellow arrowhead) but maintains robust signal in *Blm* mutant testes (example GSC yellow arrowhead).

(L, Q) 3 days post irradiation, γH2AvD signal persists (yellow arrowhead) or is diminished (yellow arrow) GSCs of control testes. 29/30 *Blm* null mutant testes contained no GSCs, 1/30 testes contained 1 GSC (Q), which retains γH2AvD signal (yellow arrowhead).

(M, R) 7 days post irradiation, γH2AvD signal is diminished throughout control testes (example GSC, yellow arrow, M). γH2AvD signal is retained in the hub of *Blm* null mutant testes and the germline is completely lost.

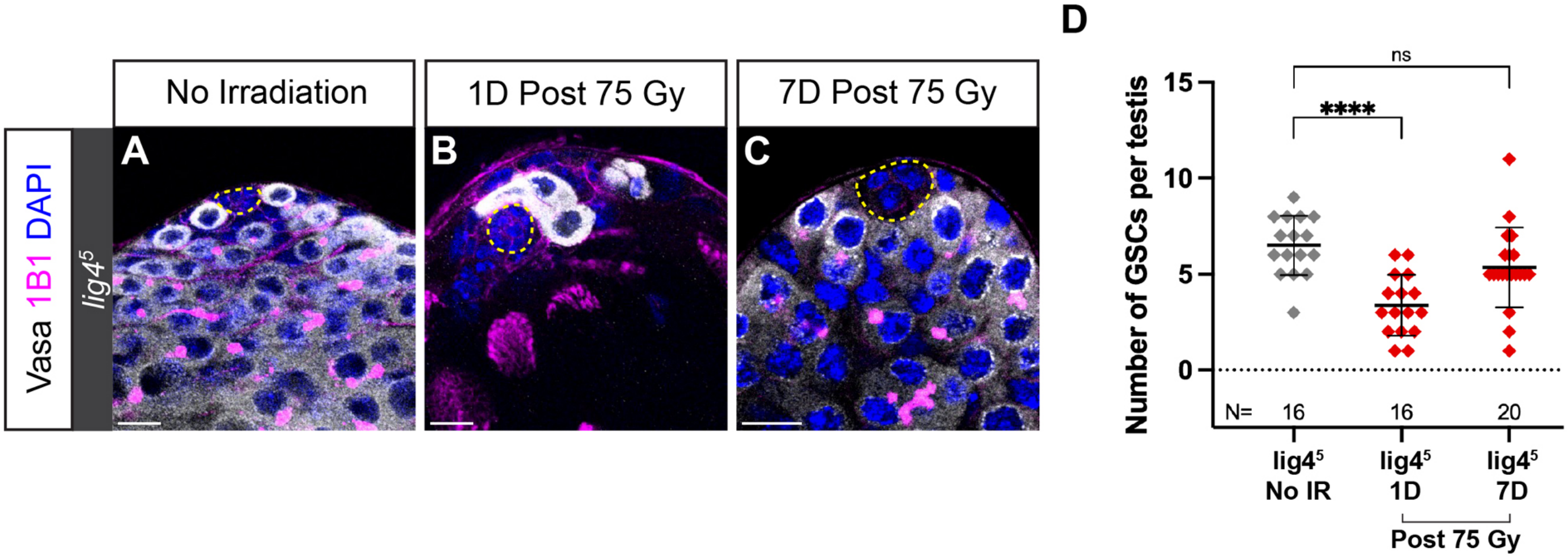

Fig. S7. DNA damage-induced GSC regeneration does not require NHEJ pathway member DNA ligase 4 (lig4).

(A-C) Confocal sections through the apex of *lig4*^{5}*/Y* testes immuno-stained with anti-Vasa (germ cells, white), anti-1B1 (fusome, magenta) and DAPI (DNA, blue). The hub is outlined in yellow. *lig4*^{5} mutant testes are with or without irradiation as indicated. The scale bar is 10 μm.

(D) Quantification of the data from (A-C) show that *lig4*^{5} mutant testes initially lose but then regain GSCs by 7 days after irradiation. ****$p<0.0001$, one way ANOVA with Tukey's multiple comparisons. Bars indicate mean and S.D. N = number of testes.

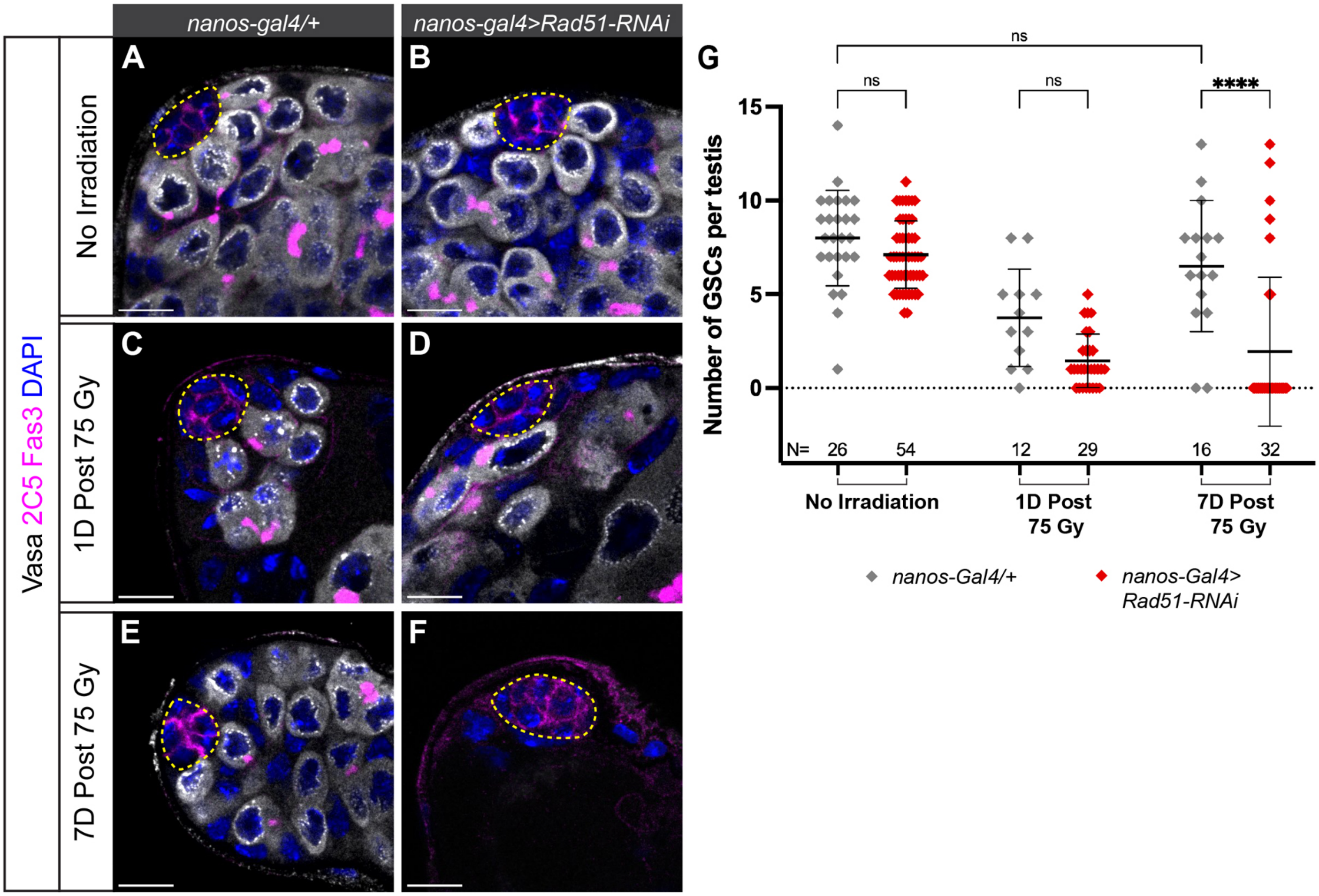

Fig. S8. There is a cell-autonomous requirement of HR pathway member, Rad51, in the germline for DNA damage induced regeneration of GSCs.

(A-F) Confocal sections through the apex of testes immuno-stained with anti-Vasa (germ cells, white), anti-2C5 (fusome, magenta), anti-Fasciclin 3 (hub cells, magenta), and DAPI (DNA, blue). The hub is outlined in yellow. Driver only control or *Rad51* knockdown testes are with or without irradiation as indicated. Scale bar is 10 µm.

(G) Quantification of the data in (A-F) showing that GSCs in control testes initially lose and then regain their GSCs. While the *nanos-Gal4>Rad51-RNAi* initially lose their GSCs and then fail to recover by 7 days post irradiation. ****p<0.0001, ordinary two-way ANOVA with Tukey's multiple comparisons. Bars indicate mean and S.D. N = number of testes.

Table S1. Key resource table

**Reagent type (species) or resource**	**Designation**	**Source or Reference**	**Identifiers**	**Additional Notes**
Genetic reagent (*D. melanogaster*)	*UASp-p35-FLAG*	Gift from Ting Xie (Ma et al., 2016)		
Genetic reagent (*D. melanogaster*)	*mnk[p6] (Chk2[p6])*	Gift from Zhao Zhang (Abdu et al., 2002)		
Genetic reagent (*D. melanogaster*)	*y w; P{GAL4::VP16-nanos.UTR} (nanos-Gal4)*	Gift from Erica Selva (Van Doren et al., 1998)		
Genetic reagent (*D. melanogaster*)	*w; nanos-GAL4 B10-2 (nanos-Gal4)*	Gift from Mark Van Doren		Affiliation: Johns Hopkins University - Krieger School of Arts and Sciences
Genetic reagent (*D. melanogaster*)	*[ru cu ca] spnA[093A]/TM3, Sb[1]*	Gift from Jan LaRocque (Staeva-Vieira et al., 2003)		
Genetic reagent (*D. melanogaster*)	*y w;ku70[7B2.2]/TM6B Tb Hu*	Gift from Jan LaRocque (Johnson-Schlitz et al., 2007)		
Genetic reagent (*D. melanogaster*)	*y w;ku70[Ex8]/TM6B Tb Hu*	Gift from Jan LaRocque (Johnson-Schlitz et al., 2007)		
Genetic reagent (*D. melanogaster*)	*P{nos::eGFP-Moe::nos 3'UTR}*	Gift from Ruth Lehmann (Sano et al., 2005)		

Development • Supplementary information

Genetic reagent (*D. melanogaster*)	*HS-FLP/Y; Tubulin > STOP > Gal4, UAS GFP*	Gift from Ben Ohlstein		Affiliation: UT Southwestern Medical Center
Genetic reagent (*D. melanogaster*)	*UAS-GFP-Moesin Actin-binding domain*	Gift from Dan Kiehart (Bloor and Kiehart, 2001; Dutta et al., 2002)		
Genetic reagent (*D. melanogaster*)	*y[1] w[1118]*	Bloomington Drosophila stock center	RRID:BDSC_6598	
Genetic reagent (*D. melanogaster*)	*Blm[D3] ry[506]/TM3, Sb[1] ry[RK]*	Bloomington Drosophila stock center	RRID:BDSC_8656	
Genetic reagent (*D. melanogaster*)	*w[1118]; Blm[N1]/TM3, Sb[1]*	Bloomington Drosophila stock center	RRID:BDSC_28878	
Genetic reagent (*D. melanogaster*)	*w[1118] DNAlig4[5]*	Bloomington Drosophila stock center	RRID:BDSC_8519	
Genetic reagent (*D. melanogaster*)	*UAS-Rad51-RNAi: y[1] sc[*] v[1] sev[21]; P{y[+t7.7] v[+t1.8]=TRiP.GL00669} attP2*	Bloomington Drosophila stock center	RRID:BDSC_38898	
Genetic reagent (*D. melanogaster*)	*UAS-Chk2-RNAi*	Bloomington Drosophila stock center	RRID:BDSC_35152	
Genetic reagent (*D. melanogaster*)	*UAS-Ku70-RNAi: y[1] v[1]; P{y[+t7.7] v[+t1.8]=TRiP.HMJ2352 6}attP40*	Bloomington Drosophila stock center	RRID:BDSC_61942	
Genetic reagent (*D. melanogaster*)	*y[1] w[1118]; P{w[+mC]=UAS-p53.R155H.Ex}2/T(2;3) TSTL, CyO: TM6B, Tb[+]*	Bloomington Drosophila stock center	RRID:BDSC_8419	
Genetic reagent (*D. melanogaster*)	*Df(3R)X3F, P{ry[+t7.2]=RP49}mtg[P 2] e[1]/MKRS*	Bloomington Drosophila stock center	RRID:BDSC_2352	
Genetic reagent (*D. melanogaster*)	*y[1] w[1118];p53[11-1B-1]*	Bloomington Drosophila stock center	RRID:BDSC_6816	

Genetic reagent (*D. melanogaster*)	*y[1] w[1118]; p53[5A-1-4]*	Bloomington Drosophila stock center	RRID:BDSC_6815	
Genetic reagent (*D. melanogaster*)	*y[1]; lok[KD]/CyO (Chk2[KD]/Cyo)*	Bloomington Drosophila stock center	RRID:BDSC_605592	
Genetic reagent (*D. melanogaster*)	*w[*]; P{w[+mC]=His2Av-mRFP1}III.1*	Bloomington Drosophila stock center	RRID:BDSC_23650	
Antibody	(Mouse monoclonal) 7G10 anti–Fasciclin III (*Drosophila*)	Developmental Studies Hybridoma Bank	Cat#7G10 anti-Fasciclin III; RRID: AB_528238	IHC (1:50)
Antibody	(Rat monoclonal) anti-Vasa	Developmental Studies Hybridoma Bank	Cat# anti-vasa; RRID: AB_760351	IHC (1:50)
Antibody	(Rabbit polyclonal) anti-Vasa	Santa Cruz Biotechnology	Cat# sc-30210; RRID: AB_793874	IHC (1:200)
Antibody	(Mouse monoclonal) anti-Hts (1B1)	Developmental Studies Hybridoma Bank	Cat# 1B1; RRID: AB_528070	IHC (1:50)
Antibody	(Mouse monoclonal) anti-Hts (2C5)	A third unpublished monoclonal raised against the same hts antigen as the published antibodies (Zaccai and Lipshitz, 1996). Gift from Howard Lipshitz		Affiliation: University of Toronto
Antibody	(Guinea Pig polyclonal) anti-zfh1	Laboratory of Erika Matunis		IHC (1:500), Affiliation: Johns Hopkins University - School of Medicine
Antibody	(Chicken polyclonal) anti-GFP	Abcam	Cat# ab13970; RRID:AB_300798	IHC (1:10,000)
Antibody	(Mouse) anti-FLAG	Sigma Life Sciences	Cat# F1804; RRID:AB_262044;	IHC (1:500)

Development • Supplementary information

Antibody	(Rabbit polyclonal) anti-Histone H2AvD phosphoS137 Antibody	Rockland	Cat# 600-401-914S; RRID:AB_828383	IHC (1:1000)
Alexa Fluor secondary antibodies	Goat anti-Chick IgY (H+L) secondary antibody, Alexa Fluor 488 conjugate	ThermoFisher Scientific	Cat# A-11039; RRID: AB_2534096	IHC (1:200)
Alexa Fluor secondary antibodies	Goat anti-Rabbit IgG (H+L) secondary antibody, Alexa Fluor 488 conjugate	ThermoFisher Scientific	Cat# A-11008; RRID: AB_143165	IHC (1:200)
Alexa Fluor secondary antibodies	Goat anti-Rabbit IgG (H+L) secondary antibody, Alexa Fluor 568 conjugate	ThermoFisher Scientific	Cat# A-11011; RRID: AB_143157	IHC (1:100)
Alexa Fluor secondary antibodies	Goat anti-Rat IgG (H + L) secondary antibody, Alexa Fluor 568 conjugate	ThermoFisher Scientific	Cat# A-11077; RRID: AB_2534121	IHC (1:100)
Alexa Fluor secondary antibodies	Goat anti-Rat IgG (H+L) Cross-Adsorbed Secondary Antibody, Alexa Fluor 488	ThermoFisher Scientific	Cat# A-11006; RRID: AB_2534070	IHC (1:200)
Alexa Fluor secondary antibodies	Goat anti-Guinea Pig IgG (H + L) secondary antibody, Alexa Fluor 488 conjugate	ThermoFisher Scientific	Cat# A-11073; RRID: AB_2534117	IHC (1:200)
Alexa Fluor secondary antibodies	Goat anti-Mouse IgG (H+L) secondary antibody, Alexa Fluor 647 conjugate	ThermoFisher Scientific	Cat# A-21235; RRID: AB_2535804	IHC (1:66)
Alexa Fluor secondary antibodies	Goat anti-Mouse IgG (H+L) Highly Cross-Adsorbed Secondary Antibody, Alexa Fluor™ 488	ThermoFisher Scientific	Cat# A-11029; RRID: AB_2534088	IHC (1:200)
Alexa Fluor secondary antibodies	Goat anti-Mouse IgG (H+L) Highly Cross-Adsorbed Secondary Antibody, Alexa Fluor™ 568	ThermoFisher Scientific	Cat# A-11031; RRID: AB_144696	IHC (1:100)

Chemical compound	4,6-diamidino-2-phenylindole (DAPI)	Millipore/Sigma (formerly Sigma-Aldrich)	Cat# 10236276001; CAS: 28718-90-3	IHC (1µg/mL)
Chemical compound	16% Paraformaldehyde (formaldehyde) aqueous solution	Electron Microscopy Sciences	Cat# 15710; CAS: 50-00-0	Fixative agent (4%)
Chemical compound	VECTASHIELD Antifade Mounting Medium	Vector Laboratories	Cat# H-1000	
Chemical compound	Goat serum	Millipore/Sigma (formerly Sigma-Aldrich)	Cat# G9023	For immunostaining (see Matunis et al., 1997)
Chemical compound	Insulin from bovine pancreas	Millipore/Sigma	Cat# I6634	For live imaging solution (see Greenspan and Matunis, 2023)
Chemical compound	Penicillin-Streptomycin (10,000 U/mL)	ThermoFisher Scientific	Cat# 15140122	For live imaging solution (see Greenspan and Matunis, 2023)
Chemical compound	Schneider's Drosophila Medium	ThermoFisher Scientific	Cat# 21720024	For live imaging solution (see Greenspan and Matunis, 2023)
Chemical compound	Poly-L-lysine hydrobromide	Millipore/Sigma	Cat# P2636	For live imaging (see Greenspan and Matunis, 2023)
Chemical compound	LysoTracker Red DND-99	ThermoFisher Scientific	Cat# L7528	
Chemical compound	Apoptag Red In Situ Apoptosis Detectinon Kit	Millipore/Sigma	Cat# S7165	
Equipment	Zeiss LSM confocals	Carl Zeiss Microscopy	https://www.zeiss.com/microscopy/us/products/light-microscopes/confocal-microscopes.html	details in 'Image acquisition' section
Equipment	Gammacell 3000 Elan irradiator with Cesium-137	Best Theratronics		

Development • Supplementary information

Software	FIJI	Schindelin et al., 2012	https://fiji.sc/	
Software	Zen	Carl Zeiss Microscopy	https://www.zeiss.com/microscopy/int/products/microscope-software/zen.html	Program for Zeiss LSM confocals
Software	Prism 10	GraphPad	http://www.graphpad.com/scientific-software/prism/	
Software	Imaris	Bitplane	http://www.bitplane.com/imaris/imaris	
Website	FlyBase.org	Öztürk-Çolak et al., 2024	release FB2025_05	We used FlyBase to find information on phenotypes, function, stocks and gene expression
Website	Biorender.com	Created in BioRender. Grey, J. (2026)	https://BioRender.com/2yhwf7k	We used Biorender to generate the diagram in Fig. 3E

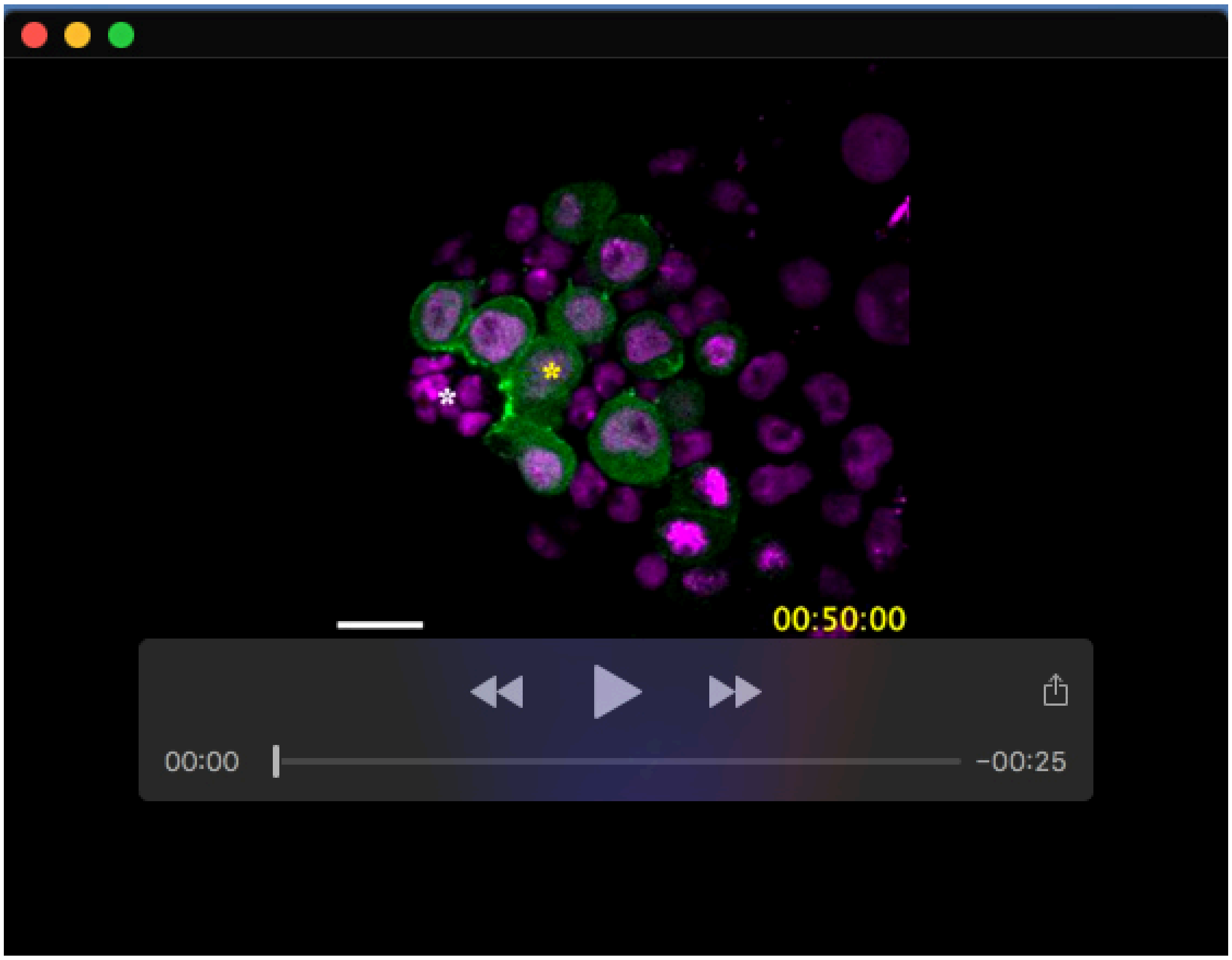

Movie 1. A single GSC detaches and displaces from the niche post 75 Gy of irradiation. Time lapse confocal sections of a testis apex live imaged starting at 3 hours post 75 Gy of irradiation (same testis as in Fig. 3A-C). Nuclei are marked with His2Av-RFP (magenta); GSCs and their daughters are marked with nanos-GMA (green). Time stamps indicate hours:minutes:seconds from the start of imaging. A single GSC (yellow asterisk) can be seen detaching and fully displacing from the hub (white asterisk) by 04:35:00; it remains displaced for the duration of the movie (12:30:00).

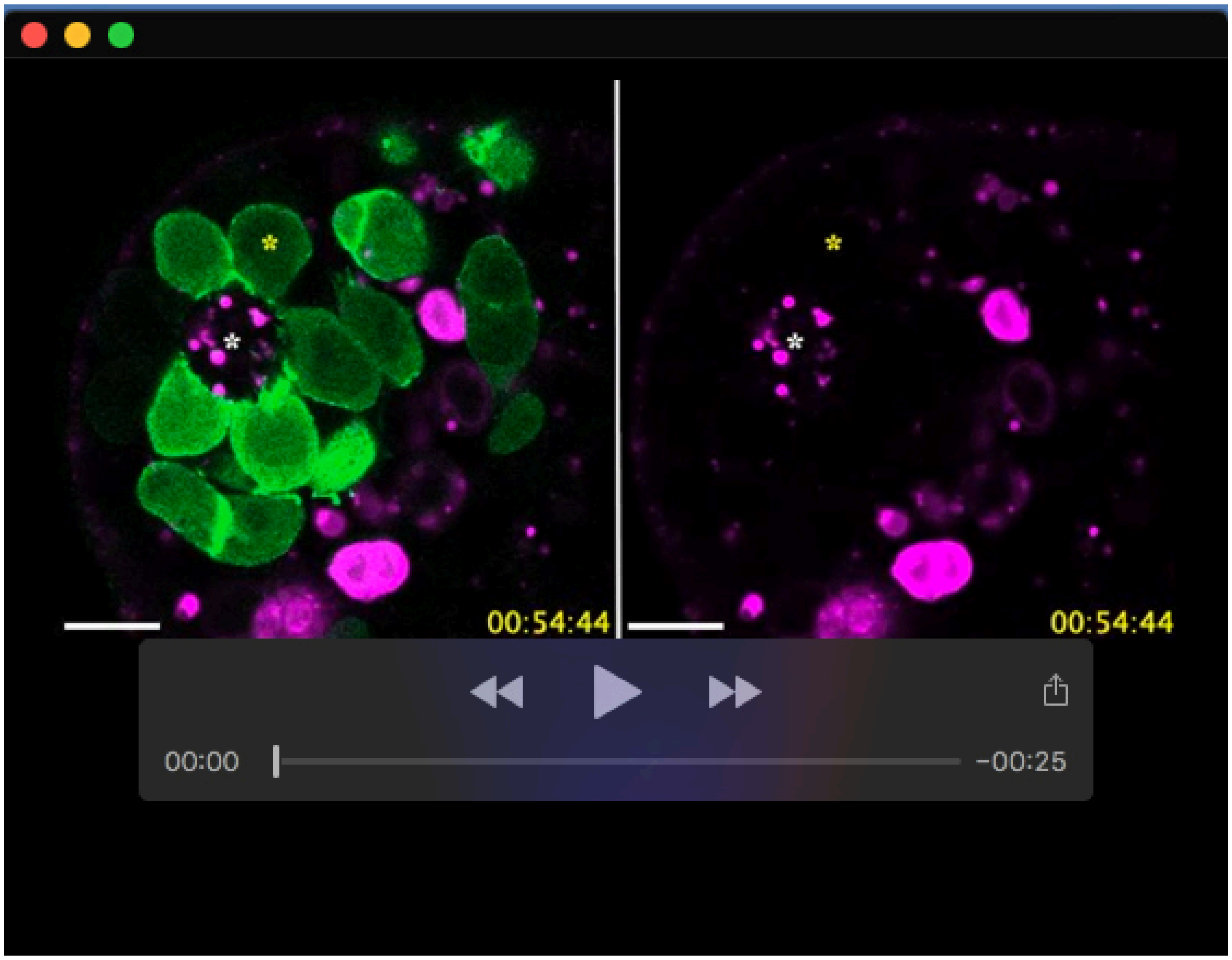

Movie 2. A single GSC detaches and displaces from the niche post 75 Gy of irradiation, divides, and later dies.

Time lapse confocal sections of a testis apex live imaged starting at 3 hours post 75 Gy of irradiation. GSCs and their daughters are marked with nanos-GMA (green). Lysotracker (magenta) marks lysosomes and dying cells. Time stamps indicate hours:minutes:seconds from the start of imaging. A single GSC (yellow asterisk), which can be seen fully detached from the hub (white asterisk) at 08:12:43, divides at 11:24:20 (daughter cell marked with a cyan asterisk), and the pair later uptakes Lysotracker beginning at 16:25:26.

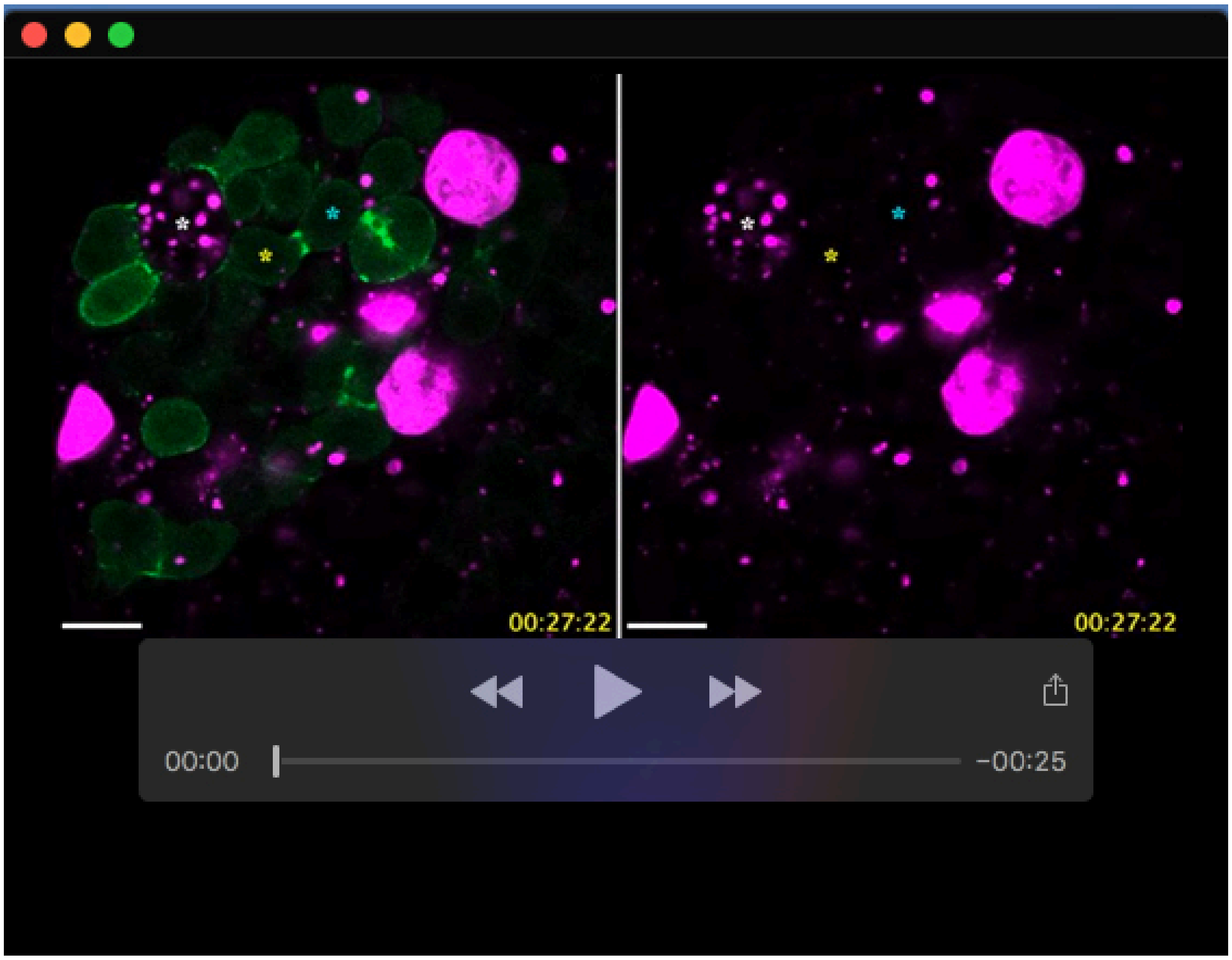

Movie 3. A GSC-daughter pair detaches and displaces from the niche post 75 Gy of irradiation and later dies.

Time lapse confocal sections of a testis apex live imaged starting at 3 hours post 75 Gy of irradiation. GSCs and their daughters are marked with nanos-GMA (green). Lysotracker (magenta) marks lysosomes and dying cells. Time stamps indicate hours:minutes:seconds from the start of imaging. A single GSC (yellow asterisk) and its attached daughter (cyan asterisk) can be seen fully detached from the hub at 09:07:28, and later uptakes Lysotracker beginning at 16:52:49.

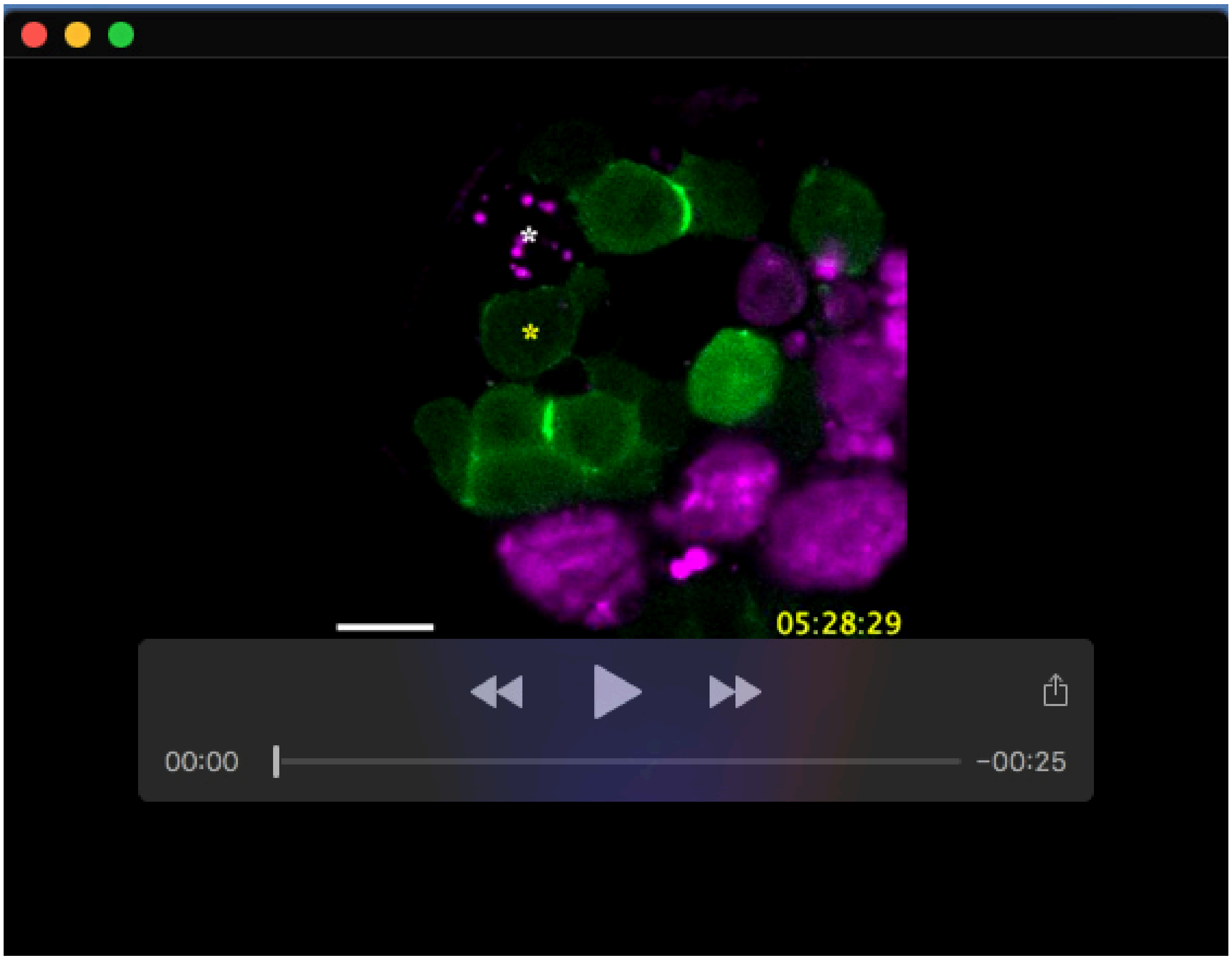

Movie 4. A GSC-daughter pair detaches and displaces from the niche post 75 Gy of irradiation and later divides.

Time lapse confocal sections of a testis apex live imaged starting at 3 hours post 75 Gy of irradiation. GSCs and their daughters are marked with nanos-GMA (green). Lysotracker (magenta) marks lysosomes and dying cells. Time stamps indicate hours:minutes:seconds from the start of imaging. A single GSC (yellow asterisk) divides to produce a daughter cell (cyan asterisk) at 06:23:14, detaches and displaces from the hub at 09:34:51, and divides into a 4-cell cyst at 17:47:34.

Development • Supplementary information

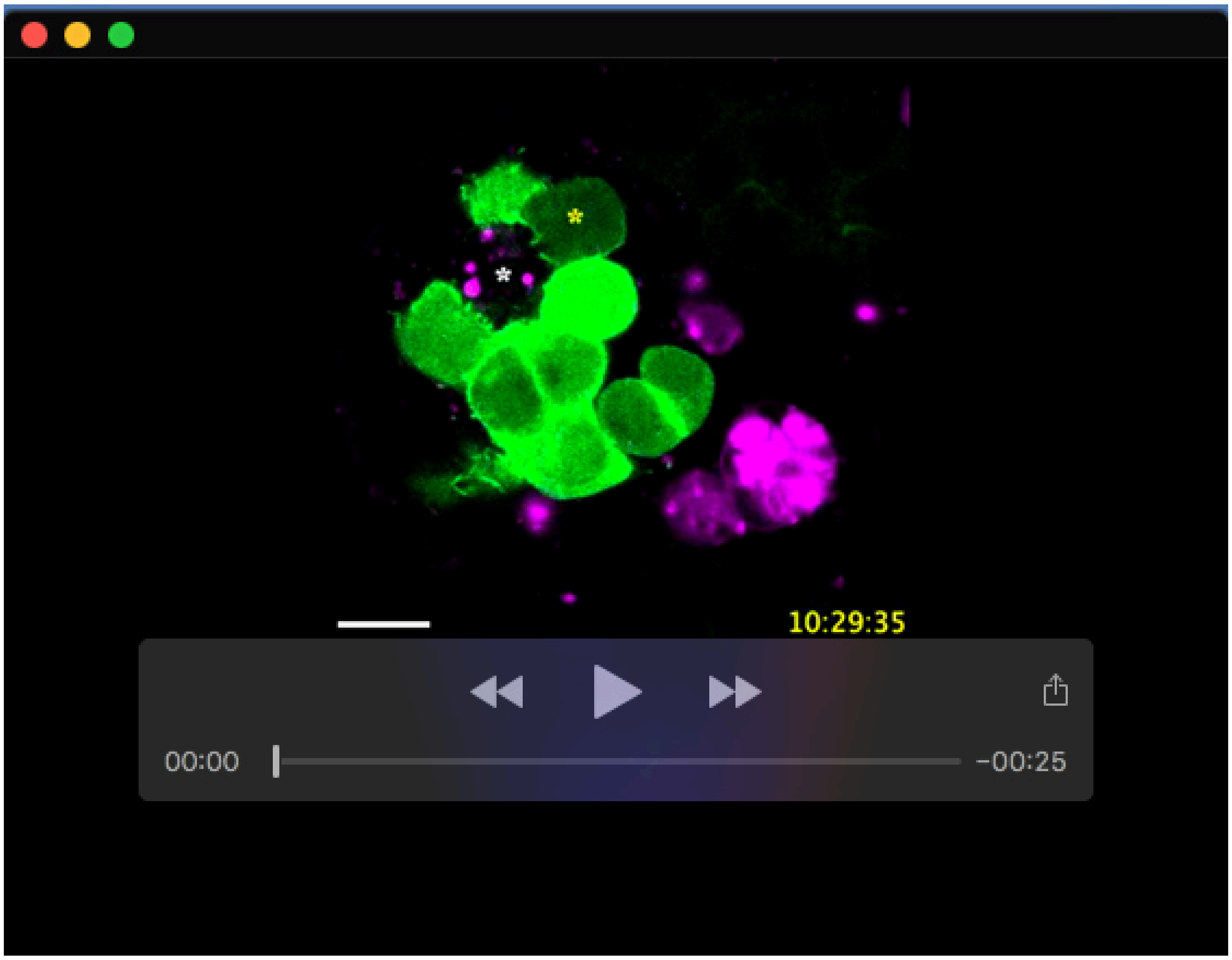

Movie 5. A GSC detaches, divides, and displaces from the niche post 75 Gy of irradiation and later returns

Time lapse confocal sections of a testis apex live imaged starting at 3 hours post 75 Gy of irradiation. GSCs and their daughters are marked with nanos-GMA (green). Lysotracker (magenta) marks lysosomes and dying cells. Time stamps indicate hours:minutes:seconds from the start of imaging. A single GSC (yellow asterisk) detaches from the hub (white asterisk) and divides into a 2-cell cyst (cyan asterisk) at 11:24:20. One germ cell of the 2-cell cyst regains contact with the hub at 17:47:34.
