# Supplementary material for: Homologous recombination is essential for DNA damage-induced regeneration of Drosophila testis germline stem cells
Source: Development. 2026 Aug 13;153(15):dev205514. doi: 10.1242/dev.205514 (PMC13535101; doi:10.1242/dev.205514)
Supplement: Supplementary file 2 [file develop-153-205514_review_history.pdf]

Homologous recombination is essential for DNA damage-induced regeneration of *Drosophila* testis germline stem cells

Jasmine R. Grey, Salman Hasan, Janelle Bellot, Margaret de Cuevas and Erika L. Matunis

DOI: 10.1242/dev.205514

Editor: Cassandra Extavour

Review timeline

Original submission: 16 January 2026
Editorial decision: 25 February 2026
First revision received: 16 June 2026
Accepted: 6 July 2026

Original submission

First decision letter

MS ID#: dev.205514

MS TITLE: Homologous recombination is essential for DNA damage-induced regeneration of Drosophila testis germline stem cells

AUTHORS: Jasmine Renée Grey, Salman Hasan, Janelle Bellot, Margaret de Cuevas and Erika L. Matunis

Dear Dr Matunis,

I have now received all the referees reports on the above manuscript, and have reached a decision. The referees' comments are appended below.

The overall evaluation is positive and we would like to publish a revised manuscript in Development, provided that the referees' comments can be satisfactorily addressed. Please attend to all of the reviewers' comments in your revised manuscript and detail them in your point-by-point response. If you do not agree with any of their criticisms or suggestions explain clearly why this is so. If it would be helpful, you are welcome to contact us to discuss your revision in greater detail. Please send us a point-by-point response indicating your plans for addressing the referees' comments, and we will look over this and provide further guidance.

Reviewer 1

The study by Grey et al. investigates the response of stem cells to ionizing radiation (IR) using the Drosophila male germline stem cells (GSCs) as a model system. This research group had previously shown that male GSCs are resistant to IR-induced apoptosis and that this resistance is regulated by Jak-Stat pathway regulation of DIAP. In this study, they use genetics with fixed and live cell imaging to show that IR induces GSCs to leave the niche. Upon recovery from IR, the niche is repopulated by GSCs. They find that the initial GSC loss and later recovery requires both p53 and Chk2 cell autonomously. While mutations in NHEJ DNA repair genes did not affect initial loss or later recovery of GSC, those in HR DNA repair genes affect GSC recovery, but not the initial GSC loss. They further showed that the initial high level of DNA repair foci after IR was resolved during recovery, and that p53 is required for this DNA repair.

© 2026. Published by The Company of Biologists under the terms of the Creative Commons Attribution License (https://creativecommons.org/licenses/by/4.0/).

This is a very well written manuscript that addresses the interesting question of how radiation response differs in stem cells. Their results reveal that the genetic requirements for male GSC loss and recovery after IR are both similar to and different from that previously described for female GSC. It is known that stem cells have altered stress responses from flies to mammals, but how these modified responses vary among stem cell types is understudied and of high medical relevance. The data is rigorous and well-presented and largely supports the conclusions. I only have one comment that may require additional data, whereas others can be addressed by editing.

1) The recovery of g-H2Av repair foci was not analyzed in NHEJ and HR mutants. If damage is repaired in NHEJ but not HR mutants, it would support the conclusion that HR but not NHEJ is required for DNA repair, and would further link the failure to repair to the failure to repopulate the niche in HR mutants. It would also suggest that p53 mutants may be repair-defective because of a failure to activate HR. This is such an obvious missing experiment that I would be surprised if the authors have not done it already.

2) In wild type, GSC are lost in early time points after IR and recover in later time points. p53 and Chk2 mutants do not have GSC loss in early time points and then have fewer GSC than wild type in later time points. This is interpreted to mean that p53 and Chk2 are required for both initial GSC loss and later recovery of GSC. Given that the mutants do not have an initial GSC loss, however, what are they expected to recover from? Instead, perhaps what the data is revealing is that there is a delayed loss of GSC in p53 and Chk2 nulls through an alternative pathway? Maybe the mutants would recover GSC in even later time points? These points can be addressed experimentally or by editing of results and discussion.
Minor:

3) Line 395:. "In the Drosophila ovary and testis, p53 expression is upregulated in the GSCs and immediate daughters after irradiation (Wylie et al., 2014)."

While there is some increase in fly p53 expression after IR, it is minimal compared to that which occurs in mammals. The Wylie et al. study did not examine p53 expression after IR, only its activity using a rpr-GFP reporter. They have called that reporter p53R-GFP in a number of papers, which has led to a lot of confusion in the field.

4) The authors may wish to consider and cite Chakravarti et al. 2022 eLife DOI: 10.7554/eLife.61389 who examined the requirement of p53 for apoptosis and DNA repair in the Drosophila female germline. That study also showed that female GSC and meiotic cells are resistant to IR.

5) Line 411: Although in the ovary p53 is not responsible for GSC loss after DNA damage, it contributes to GSC preservation over time (Ma et al., 2016).Thus, p53 plays a role in both the ovary and the testis in maintaining GSCs in the stem.

6) Line 421 "shows" change to "showed"

7) line 426 "Together, this poses an interesting question as to why the GSCs in the testis fail to use NHEJ upon DNA damage when HR is unavailable, as the mechanisms that ensure repair occurs by HR in GSCs are an important focus for future work."

Seems like there is a logic link issue between clauses?

8) Line 255 "cell-intrinsically" Is that different than the standard term of cell autonomously?

9) Cite FlyBase: Ozturk-Colak A, et al. 2024. Genetics. 10.1093/genetics/iyad211

<u>Reviewer 2</u>

SUMMARY OF THE ADVANCE MADE IN THIS PAPER AND ITS POTENTIAL SIGNIFICANCE TO THE FIELD

© 2026. Published by The Company of Biologists under the terms of the Creative Commons Attribution License (https://creativecommons.org/licenses/by/4.0/).

This manuscript describes the detailed characterization of how cells within the Drosophila testis respond to irradiation. The authors start by showing a reversible reduction of germline stem cells (GSCs) adjacent to the hub upon exposure to 75 Gy of irradiation. This loss is not caused by apoptosis. Rather GSCs leave the niche as single cells. Interestingly, GSC numbers increase after 7 days post irradiation. The ability of GSCs to leave the niche and then move back to the hub after 7 days requires Chk2, p53, Blm and Rad51, the last two genes indicating a functional role for the homologous recombination repair pathway. By contrast, Ku70, a component of the c-NHEJ pathway, is not required for this behavior. This study establishes the testis as a robust model for studying DNA repair pathway choice in vivo and provides compelling evidence that HR dependent repair mechanisms promote the ability of GSCs to regenerate after irradiation.

SUGGESTIONS TO AUTHORS

This is a strong study that will be of interest to the readership of Development. The text is well-written, and the presented data generally support the conclusions made by the authors. A couple of points should be addressed before publication of the work.

1) In figure 4, the authors use only one allele of Chk2 and p53 in their analysis. Additional alleles and/or placing the mutations over a deficiency would reduce the likelihood that other unlinked mutations on the chromosome contribute to the observed phenotypes. A stronger experiment would be to perform rescue experiments with transgenes, if available. These concerns are somewhat mitigated by the RNAi/dominant negative experiments presented in Figure S3. However, at the 7-day time point, neither the knockdown of Chk2 or the expression of the p53-R155H transgene are statistically different from the controls. Shoring up the genetics of Chk2 and p53 will strength the conclusions of the paper.
2) The accompanying movies nicely illustrate the behaviors of cells within the testis after irradiation. However, movie 5, which is supposed to capture a germ cell moving back into the niche, ends shortly after the cell touches the hub. To my eye, re-association occurs at 17:47:34 and lasts until the end of the movie (18:42:19). Is this correct? Providing annotations to the movies may help direct the reader. Do the authors believe this association is stable over days? I recognize making these movies is technically difficult, but can the authors include additional examples of this behavior?
3) Are the germ cells that re-associate with the niche 7 days after exposure to 75 Gy irradiation functional? In other words, do the authors have evidence they produce gonialblasts that go on to differentiate into functional sperm?

First revision

<u>Author response to reviewers' comments</u>

We thank the reviewers for their very helpful comments, which have led us to improve our work through both the completion of new experiments and by modifications to the text. We have added two new supplementary figures and have made several revisions to the results and discussion, as detailed in our point-by point response below. Reviewers' comments are in Roman text, authors' responses are italicized.

Reviewer 1: The study by Grey et al. investigates the response of stem cells to ionizing radiation (IR) using the Drosophila male germline stem cells (GSCs) as a model system. This research group had previously shown that male GSCs are resistant to IR-induced apoptosis and that this resistance is regulated by Jak-Stat pathway regulation of DIAP. In this study, they use genetics with fixed and live cell imaging to show that IR induces GSCs to leave the niche. Upon recovery from IR, the niche is repopulated by GSCs. They find that the initial GSC loss and later recovery requires both p53 and Chk2 cell autonomously. While mutations in NHEJ DNA repair genes did not affect initial loss or later recovery of GSC, those in HR DNA repair genes affect GSC recovery, but not the initial GSC loss. They further showed that the initial high level of DNA repair foci after IR was resolved during recovery, and that p53 is required for this DNA

© 2026. Published by The Company of Biologists under the terms of the Creative Commons Attribution License (https://creativecommons.org/licenses/by/4.0/).

repair.

This is a very well written manuscript that addresses the interesting question of how radiation response differs in stem cells. Their results reveal that the genetic requirements for male GSC loss and recovery after IR are both similar to and different from that previously described for female GSC. It is known that stem cells have altered stress responses from flies to mammals, but how these modified responses vary among stem cell types is understudied and of high medical relevance. The data is rigorous and well-presented and largely supports the conclusions. I only have one comment that may require additional data, whereas others can be addressed by editing.

1) The recovery of g-H2Av repair foci was not analyzed in NHEJ and HR mutants. If damage is repaired in NHEJ but not HR mutants, it would support the conclusion that HR but not NHEJ is required for DNA repair, and would further link the failure to repair to the failure to repopulate the niche in HR mutants. It would also suggest that p53 mutants may be repair-defective because of a failure to activate HR. This is such an obvious missing experiment that I would be surprised if the authors have not done it already.

*Thank you for suggesting this experiment. In response, we acquired new data examining the recovery of repair foci in NHEJ (*Ku70*) or HR (*Blm*) null mutants (Fig. S6, and on lines 316-318 and 342-345). Results in *Ku70* mutant flies were straightforward. As expected, by 1 day post irradiation, *Ku70* null and control GSCs had varied levels of gamma-H2AvD signal (a proxy for the presence of DNA double-stranded breaks). By 7 days post irradiation, the gamma-H2AvD signal was fully resolved in both genotypes. These new data are consistent with our prior finding that GSCs do not require NHEJ, and links the ability of *Ku70* null GSCs to repair DSBs with their ability to repopulate the niche after IR.*

*In contrast, by 7 days post-IR, *Blm* null flies had lost all GSCs, precluding analysis of repair foci in these cells. We attempted to look at an earlier time point post-IR, but even by 3 days post-IR, only one of 30 testes retained any germ cells (in this case, a single germ cell) within the niche (Fig. S6 and lines 342-345).*

Interestingly, this sole remaining germ cell contained g-h2avd repair foci, but our current conditions do not yield conclusive data regarding a connection between the lack of Blm and the persistence of unresolved repair foci. If conditions exist wherein the loss of Blm-deficient GSCs from the niche occurs after the resolution of repair foci in control testes, we can answer this straightforward yet challenging question in the future.

2) In wild type, GSC are lost in early time points after IR and recover in later time points. p53 and Chk2 mutants do not have GSC loss in early time points and then have fewer GSC than wild type in later time points. This is interpreted to mean that p53 and Chk2 are required for both initial GSC loss and later recovery of GSC. Given that the mutants do not have an initial GSC loss, however, what are they expected to recover from? Instead, perhaps what the data is revealing is that there is a delayed loss of GSC in p53 and Chk2 nulls through an alternative pathway? Maybe the mutants would recover GSC in even later time points? These points can be addressed experimentally or by editing of results and discussion.

*We agree that stem cell recovery in the absence of loss is illogical, and that loss of GSCs in p53 and/or Chk2 nulls may be delayed or happen through alternate pathways, and we have modified the text accordingly on (lines 281-287) We also include new data regarding this topic, as described **below** in our response to reviewer 2 (Fig. S3 and lines 246-251, 260-262, 279-285).*

Minor:

3) Line 395:. "In the Drosophila ovary and testis, p53 expression is upregulated in the GSCs and immediate daughters after irradiation (Wylie et al., 2014)."

While there is some increase in fly p53 expression after IR, it is minimal compared to that which occurs in mammals. The Wylie et al. study did not examine p53 expression after IR, only its activity using a rpr-GFP reporter. They have called that reporter p53R-GFP in a number of

© 2026. Published by The Company of Biologists under the terms of the Creative Commons Attribution License (https://creativecommons.org/licenses/by/4.0/).

papers, which has led to a lot of confusion in the field.

Thank you for helping us clarify this point. We agree that Wylie et al.'s use of reaper-GFP transgene expression as a reporter of p53 activity is entirely different from p53 expression. We are adjusting our wording on Line 417 accordingly.

4) The authors may wish to consider and cite Chakravarti et al. 2022 eLife DOI: 10.7554/eLife.61389 who examined the requirement of p53 for apoptosis and DNA repair in the Drosophila female germline. That study also showed that female GSC and meiotic cells are resistant to IR.

Thank you for bringing this paper to our attention, it is now cited (line 432).

5) Line 411: Although in the ovary p53 is not responsible for GSC loss after DNA damage, it contributes to GSC preservation over time (Ma et al., 2016). Thus, p53 plays a role in both the ovary and the testis in maintaining GSCs in the stem.

Here the reviewer copied our text but did not ask a question, so we were not sure what needed to be addressed.

6) Line 421 "shows" change to "showed"

This change has been made to line 440.

7) line 426 "Together, this poses an interesting question as to why the GSCs in the testis fail to use NHEJ upon DNA damage when HR is unavailable, as the mechanisms that ensure repair occurs by HR in GSCs are an important focus for future work."

Seems like there is a logic link issue between clauses?

We agree. This statement now reads: "Overall, our work poses an interesting question as to why the GSCs in the testis fail to use NHEJ upon DNA damage when HR is unavailable. The underlying mechanisms that ensure repair occurs by HR in GSCs are an important focus for future work." *(line 446)*

8) Line 255 "cell-intrinsically" Is that different than the standard term of cell autonomously?
The term "cell-intrinsically" is not different from the standard term and has now reads "cell autonomously" (line 266).

9) Cite FlyBase: Ozturk-Colak A, et al. 2024. Genetics. 10.1093/genetics/iyad211

Thank you for bringing this important citation to our attention. It is now in the references and cited (line 493).

Reviewer 2:

SUMMARY OF THE ADVANCE MADE IN THIS PAPER AND ITS POTENTIAL SIGNIFICANCE TO THE FIELD

This manuscript describes the detailed characterization of how cells within the Drosophila testis respond to irradiation. The authors start by showing a reversible reduction of germline stem cells (GSCs) adjacent to the hub upon exposure to 75 Gy of irradiation. This loss is not caused by apoptosis. Rather GSCs leave the niche as single cells. Interestingly, GSC numbers increase after 7 days post irradiation. The ability of GSCs to leave the niche and then move back to the hub after 7 days requires Chk2, p53, Blm and Rad51, the last two genes indicating a functional role for the homologous recombination repair pathway. By contrast, Ku70, a component of the c-NHEJ pathway, is not required for this behavior. This study establishes the testis as a robust model for studying DNA repair pathway choice in vivo and provides compelling evidence that HR dependent repair mechanisms promote the ability of GSCs to regenerate after irradiation.

© 2026. Published by The Company of Biologists under the terms of the Creative Commons Attribution License (https://creativecommons.org/licenses/by/4.0/).

SUGGESTIONS TO AUTHORS

This is a strong study that will be of interest to the readership of Development. The text is well-written, and the presented data generally support the conclusions made by the authors. A couple of points should be addressed before publication of the work.

1) In figure 4, the authors use only one allele of Chk2 and p53 in their analysis. Additional alleles and/or placing the mutations over a deficiency would reduce the likelihood that other unlinked mutations on the chromosome contribute to the observed phenotypes. A stronger experiment would be to perform rescue experiments with transgenes, if available. These concerns are somewhat mitigated by the RNAi/dominant negative experiments presented in Figure S3. However, at the 7-day time point, neither the knockdown of Chk2 or the expression of the p53-R155H transgene are statistically different from the controls. Shoring up the genetics of Chk2 and p53 will strength the conclusions of the paper.

We agree and have addressed this experimentally by using two additional genotypes to investigate the requirement of Chk2 *and* p53 *post irradiation.*

For Chk2*, we selected a loss of function allele that results in a kinase dead* Chk2 *called* $Chk2^{KD}$*. We crossed* $Chk2^{KD}$ *to the* Chk2 *null allele we used previously* ($Chk2^{p6}$)*. Similar to* $Chk2^{p6}$ *homozygotes, this new allelic combination showed no loss of GSCs one day post IR, confirming that GSC loss does not occur by this time point (Fig. S3, lines 246-251; see also our response to Reviewer 1). At 7 days post-IR, although GSC numbers were reduced in the original* $Chk2^{p6}$ *homozygotes, their number did not change significantly in* $Chk2^{KD}$/ $Chk2^{p6}$ *males. We have modified our interpretation of the results at the 7-day time point, noting that loss of GSCs in Chk2 nulls may be delayed or happen through alternate pathways (lines 281-287). For an additional* p53 *allele we selected an independently generated loss of function allele,* $p53^{11\text{-}1B\text{-}1}$, and *crossed it to the* $p53^{5A\text{-}1\text{-}4}$ *null allele we used previously, generating* $p53^{11\text{-}1B\text{-}1}/p53^{5A\text{-}1\text{-}4}$*. Data from this new genotype phenocopied our initial results obtained with* $p53^{5A\text{-}1\text{-}4}$ *homozygotes: GSCs were maintained 1 day post irradiation, and were then significantly reduced by 7 days post irradiation (Fig. S3 and lines 260-262 and 280-281 in the text). These results strengthen our conclusion that GSCs lacking Chk2 and p53 are unable to respond to (and therefore repair) DNA damage and are eventually lost from the niche.*

2) The accompanying movies nicely illustrate the behaviors of cells within the testis after irradiation. However, movie 5, which is supposed to capture a germ cell moving back into the niche, ends shortly after the cell touches the hub. To my eye, re-association occurs at 17:47:34 and lasts until the end of the movie (18:42:19). Is this correct? Providing annotations to the movies may help direct the reader. Do the authors believe this association is stable over days? I recognize making these movies is technically difficult, but can the authors include additional examples of this behavior?

Thank you for appreciating the movies. We agree with your assessment of the cell behavior in movie 5 and now provide annotations to the images of all movies. The association of the GSC shown in movie 5 may or may not be stable over days. Since our current live imaging approach only extends to 24 hours - there is no way to evaluate this using our current tools. A statement to this effect has been added to the text on line 393. Generating additional movies is part of our ongoing work, with optimization of our acquisition and analysis pipeline a top priority.

3) Are the germ cells that re-associate with the niche 7 days after exposure to 75 Gy irradiation functional? In other words, do the authors have evidence they produce gonialblasts that go on to differentiate into functional sperm?

Thank you for the interesting question regarding germ cell functionality post-irradiation. Direct evidence that GSCs that re-associate with the niche go on to produce functional sperm is lacking. A limitation is the inability to distinguish the progeny of re-associating germ cells

© 2026. Published by The Company of Biologists under the terms of the Creative Commons Attribution License (https://creativecommons.org/licenses/by/4.0/).

arising post-irradiation from the progeny of germ cells that were present originally. We have added a statement about this point to the text on line 464.

Parenthetically, the question of germ cell functionality after re-association with the niche arose during our original discovery of spermatogonial dedifferentiation (PMID 15143218). In this instance, the disappearance and re-appearance of spermatogonia supported the conclusion that re-associated (new) GSCs can produce differentiating daughter cells (spermatocytes); whether they produce sperm is unanswered.

Second decision letter

MS ID#: dev.205514R1

MS TITLE: Homologous recombination is essential for DNA damage-induced regeneration of Drosophila testis germline stem cells

AUTHORS: Jasmine Renée Grey, Salman Hasan, Janelle Bellot, Margaret de Cuevas and Erika L. Matunis

Dear Dr Matunis,

I am happy to tell you that your manuscript has been accepted for publication in Development, pending our standard publication integrity checks. It was accepted on 06 Jul 2026. I believe that Reviewer 1's question and suggestion are sensible, and request that you consider addressing their comment and/or adopting their suggestion, but I leave this to your discretion, and wish to be clear that this is not a requirement for acceptance of your manuscript.

Reviewer 1

I think that the authors did an outstanding job revising the manuscript in response to previous reviews. I commend the authors for performing additional experiments to analyze DNA repair in NHEJ and HR mutants.

Reviewer 2

The authors have addressed my previous concerns. I find the manuscript acceptable for publication.

© 2026. Published by The Company of Biologists under the terms of the Creative Commons Attribution License (https://creativecommons.org/licenses/by/4.0/).
